# Symptoms of systemic lupus erythematosus are diagnosed in leptin transgenic pigs

**DOI:** 10.1371/journal.pbio.2005354

**Published:** 2018-08-31

**Authors:** Junchen Chen, Weiqi Zeng, Weirong Pan, Cong Peng, Jianglin Zhang, Juan Su, Weihu Long, Heng Zhao, Xiaoxia Zuo, Xiaoyun Xie, Jing Wu, Ling Nie, Hong-Ye Zhao, Hong-Jiang Wei, Xiang Chen

**Affiliations:** 1 Department of Dermatology, Xiangya Hospital, Central South University, Changsha, China; 2 State Key Laboratory for Conservation and Utilization of Bio-Resources in Yunnan, Yunnan Agricultural University, Kunming, China; 3 Department of Rheumatology, Xiangya Hospital, Central South University, Changsha, China; 4 Department of Endocrinology, Xiangya Hospital, Central South University, Changsha, China; 5 Department of Hematology, Xiangya Hospital, Central South University, Changsha, China; 6 College of Veterinary Medicine, Yunnan Agricultural University, Kunming, China; The Scripps Research Institute, United States of America

## Abstract

Leptin is a well-known adipokine that plays a critical role in immune responses. To further explore the immunological roles of leptin, we developed a transgenic leptin pig controlled by the pig leptin (pleptin) promoter to overexpress leptin. Symptoms typically associated with systemic lupus erythematosus (SLE) were evident in this transgenic pig strain, including anemia, leukopenia, and thrombocytopenia as well as kidney and liver impairment. Histologically, there were increased immunoglobulin G (IgG) levels, elevated antiplatelet antibody (APA) levels, and deposition of immune complexes in the kidney and liver. In addition, anti-double-stranded DNA antibodies (dsDNAs), antinuclear antibodies (ANAs), and antinucleosome antibodies (ANuAs) were all significantly increased in serum immunological examinations. These findings were also accompanied by repression of the regulatory T cell (Treg) ratio. Significantly, glucocorticoid experimental therapies partially relieved the autoimmune responses and bleeding symptoms observed in these transgenic leptin pigs. Together, these results indicate that leptin plays a critical role in the development of autoimmune disorders and demonstrate that our transgenic leptin pigs can act as a valuable model of SLE.

## Author summary

Obesity is associated with many human diseases, including autoimmune diseases, metabolic syndrome, and inflammation. Leptin is one of the most thoroughly investigated cell-signaling molecules secreted by the fat tissues and is typically elevated in patients diagnosed with lupus (systemic lupus erythematosus [SLE]), an autoimmune disease in which the body’s immune system attacks healthy tissues in many organs. Studies using leptin-deficient mice have also demonstrated that leptin is involved in modulating immune cells. However, transgenic leptin mice do not develop SLE-like symptoms, which is probably due to differences between the immune systems of humans and mice. We show that, consistent with the human data, our transgenic leptin pig model develops a number of pathophysiological changes with multiorgan injuries resembling SLE. Our work supports an association between leptin and SLE-like symptoms as observed in human patients and implies a crucial role for leptin in promoting the development of SLE.

## Introduction

Systemic lupus erythematosus (SLE) is a type of complex disease that can be caused by both genetic and environmental factors [1]. The incidence rate for this disease varies between 2.9–5.1/100,000 per year, and the prevalence rate varies from 26.2–52.2/100,000 per year [2]; however, these rates are dependent upon the type of population being studied and can be influenced by age, gender, and racial background. Multiple genomic loci have been reported to be associated with SLE [3–7], and various environmental factors, such as obesity, sunlight, infection, and exogenous estrogen, have also been shown to contribute to its development [8].

Leptin is one of the most thoroughly investigated adipokines and is typically elevated in patients diagnosed with SLE. Studies using leptin-deficient mice have also demonstrated that leptin plays a role in modulating immune cell homeostasis under pathophysiological conditions [9]. However, SLE-like symptoms were not found in transgenic leptin mice, which is likely due to differences between the immune systems of humans and mice [10]. Thus, there is a need for better model systems to further identify the roles of leptin in SLE.

The role of leptin in immunology has also been extensively studied, specifically in T-cell activation and differentiation [11]. Leptin has been shown to decrease the proliferation and suppress the activity of regulatory T cells (Tregs) [12] while also increasing the proliferation of naïve T cells. Additionally, it promotes the switch towards a helper T (Th)1-cell immune response and stimulates the survival of thymic cells [13]. Recent studies have revealed that neutrophils play an important role in autoimmune diseases and that leptin also mediates their migration and metabolism as well as inhibiting their apoptosis, among other roles [14].

There is a rapidly increasing number of biomedical pig models that have been developed and published in recent years, including those used for cancer, diabetes, cystic fibrosis, and cardiovascular, neurodegenerative, immunological, and ophthalmological diseases. Because of a similarity between human and pig immune reactions, transgenic porcine models have been utilized to mimic human autoimmune diseases [15]. Cystic fibrosis is the first human genetic disease to benefit from the directed engineering of three different species of animal models, including mice, pigs, and ferrets [16]. A pig model with an interleukin-2 receptor subunit-gamma (*IL2RG*) deletion was successfully developed with T cells and natural killer (NK) cells in the absence of a thymus [17] in order to mimic human Severe Combined Immunodeficiency (SCID). Furthermore, genetically modified pigs can complement and extend disease modeling beyond transgenic mice, particularly for diseases with slowly progressing pathophysiology [18].

In the SLE-like leptin mouse model, the production of autoantibodies were reported, but other major symptoms in human patients cannot be observed in mice [19]. However, SLE patients demonstrate more complicated clinical patterns; thus, there is no single symptom/molecular marker that can independently serve as a “gold standard” for SLE diagnosis, and it is recommended by the guidelines for diagnosis and treatment of SLE to examine a combination of multiple markers [20]. The transgenic leptin pigs satisfy these guidelines for characterizing human SLE. Additionally, leptin pigs present time-dependent progressive SLE similar to what is observed in human patients, including initial erythema and multiple-organ damage as the disease progresses over time. These two aspects of the leptin pig model highlight its value for preclinical research of SLE above mouse models. One way that this model may be utilized in the future is for molecular profiling in the different stages of SLE in order to optimize therapeutic strategies throughout the disease progression.

## Results

### Generation of leptin transgenic pigs

**Fig 1:**
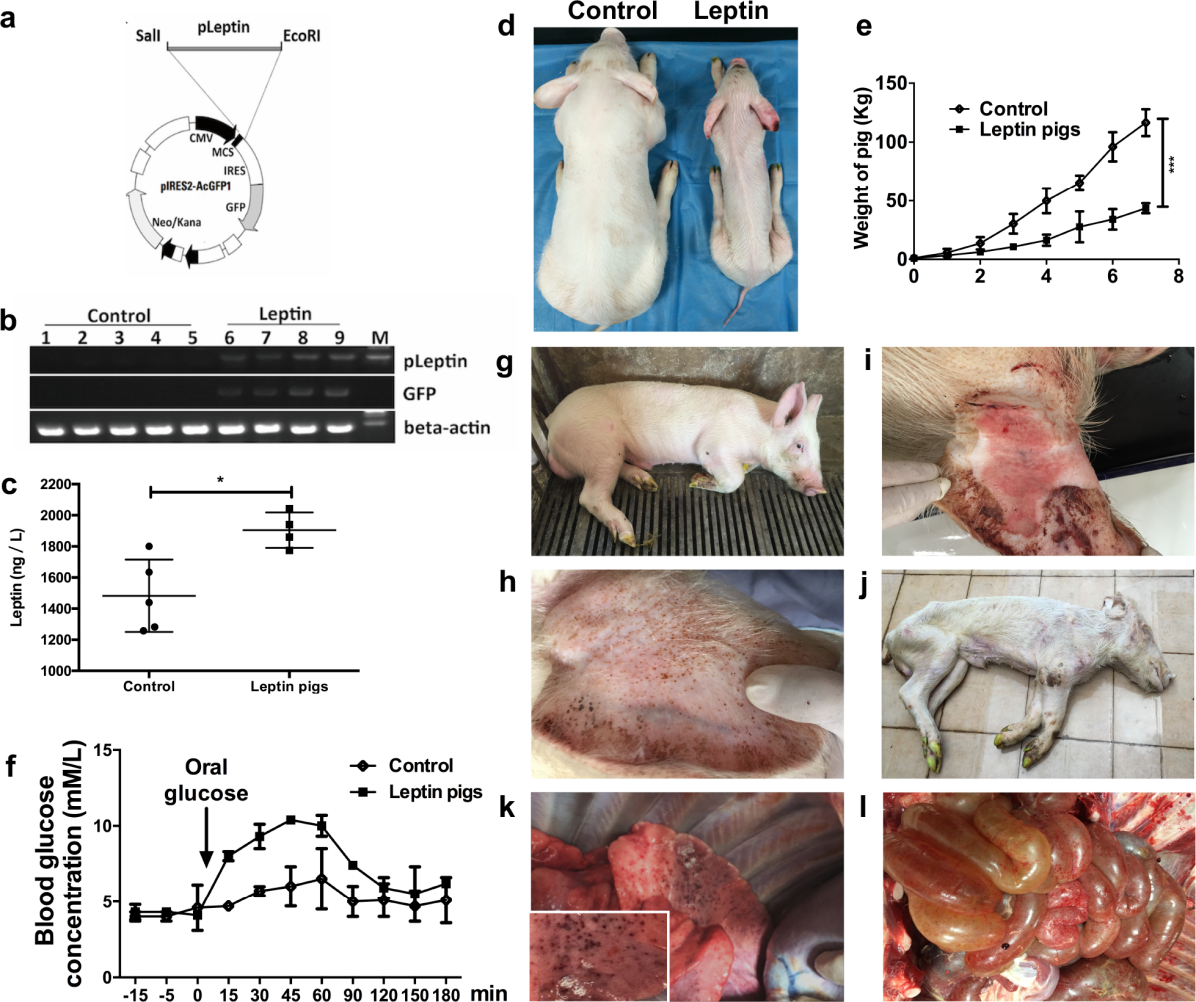
The strategy for generating transgenic leptin pigs. (a) Vector construction strategy. (b) Semiquantitative PCR results to separate leptin and control pigs. M, DNA ladder. (c) Serum leptin levels (*n* = 5 per group). (d,e) Appearances and differences in body weight in leptin and control pigs (*n* = 6 per group). (f) OGTTs reflect compromised glucose homeostasis in transgenic leptin pigs (*n* = 6 per group). (g–j) Disease development from 6 months to 8 months of age. Pigs died at 8 months old. (k–l) Anatomy of the transgenic leptin pigs when they died. *p < 0.05, ***p < 0.001, two-tailed Student *t* test for c, one-way analysis of variance for f and e. Data are presented as mean ± SEM. Numerical values are provided in S1 Data. CMV, cytomegalovirus; GFP, green fluorescent protein; IRES, internal ribosome entry sites; MCS, multiple cloning site; OGTT, oral glucose tolerance test.

The somatic cell nuclear transfer (SCNT) technique was used to generate transgenic leptin pigs (Fig 1A). Specifically, the pig leptin (pleptin) open reading frame (ORF) was amplified from a cDNA library generated from mRNA purified from a fetal pig brain and inserted into pMD18-T with a leptin promoter using the Original TA Cloning. This construct was then transfected into the fetal fibroblasts, and the leptin-positive cells were selected for SCNT (S1 Protocol). Transgenic leptin pigs (*n* = 6) were observed for up to six months of age in order to identify the immunological roles of leptin. Nontransgenic pigs served as controls (Fig 1B). The expression of leptin was verified by immunohistochemistry (IHC) (S1 Fig, upper panel), and serum leptin levels were determined by ELISA. The expression of transgenic leptin was confirmed as well (Fig 1C). To further identify the activation of leptin signaling pathways, phospho-STAT3, a transcription factor downstream of leptin, was measured [21]. Up-regulation of phospho-STAT3 confirmed the activation of leptin signaling after transgenic leptin expression (S1 Fig, lower panel). Other markers, including ERK2, STAT3, JAK2, and STAT5, were also measured to demonstrate the activation of leptin signaling in the transgenic leptin pigs (S2A and S2B Fig).

### Transgenic leptin pigs exhibit small body sizes and subcutaneous bleeding

To identify the phenotype of the transgenic leptin pigs, all pigs were physically examined twice a week. Specifically, body weights were measured, and behaviors were observed. We found that the transgenic leptin pigs had significantly lower body weights than the control pigs (34 ± 8.8 kg versus 96.1 ± 12.4 kg, *p* < 0.001) at 6 months of age (Fig 1D and 1E). Because leptin is crucial for glucose metabolism, we measured blood glucose levels in all pigs. Although basal glucose levels were similar between the controls and the transgenic leptin pigs, oral glucose tolerance tests (OGTTs) demonstrated a greater increase in blood glucose level after glucose stimulation in the leptin pigs versus the controls. Specifically, the blood glucose level increased to 8 mM/L within 15 minutes and was sustained at this level for more than 1 hour. In contrast, the control pigs responded to glucose stimulation with a slight increase in blood glucose concentration after 30 minutes, which quickly returned to baseline (Fig 1F). Leptin has also been shown to suppress insulin and thus impact glucose homeostasis in both mice and humans. Therefore, we detected insulin levels in circulating blood and found that there was a statistically significant reduction of insulin levels in the leptin pigs compared to controls (S2C Fig). These results confirmed the role of leptin in glucose metabolism in the transgenic pig model and indicated that it may be involved in the underlying mechanism behind the observed smaller body sizes and slower growth rates in the transgenic leptin pigs.

**Fig 2:**
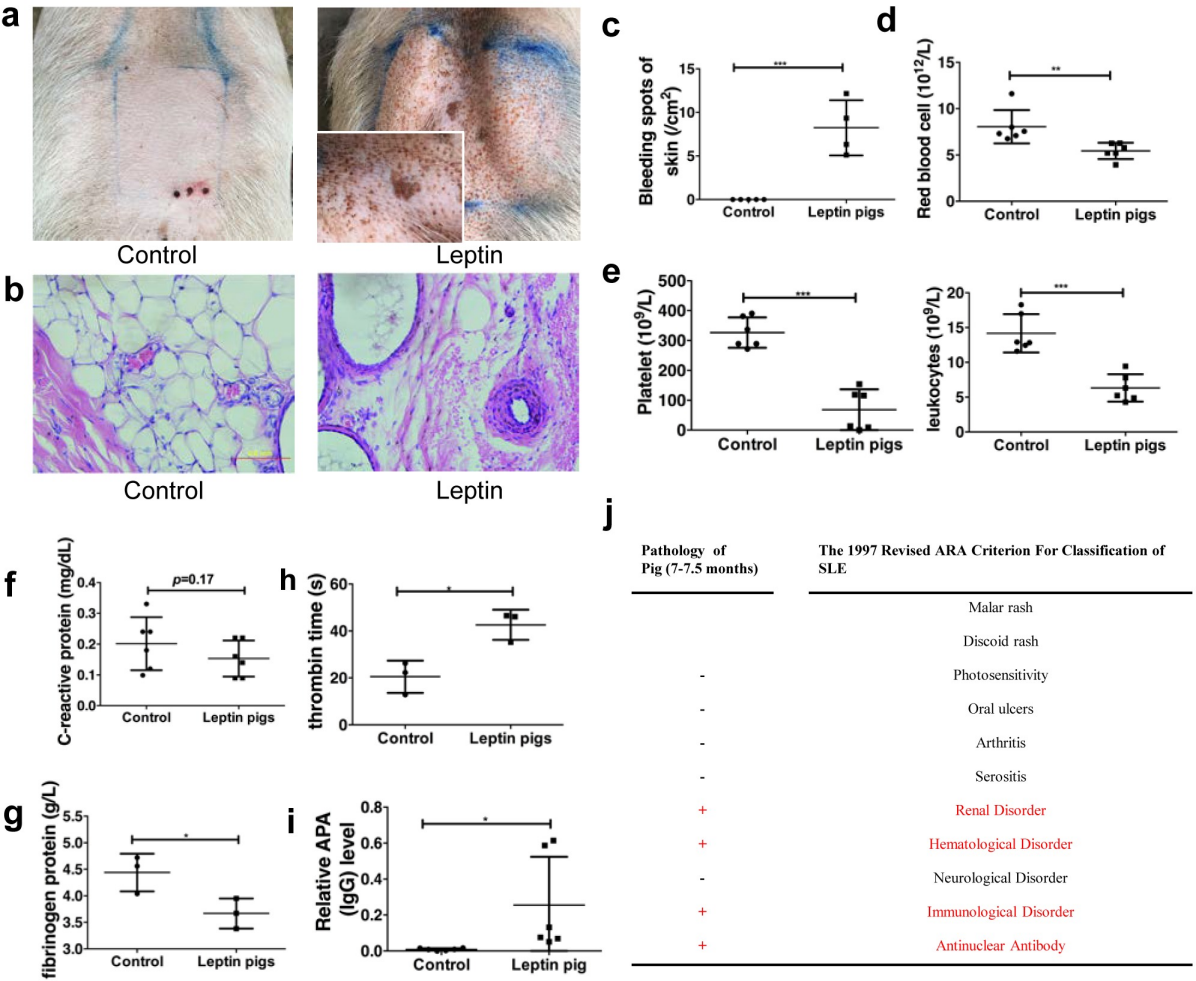
Physiological examination of leptin-overexpressing pigs. (a) Images of the backs of control and leptin-overexpressing pigs. (b) H&E staining showed RBC diffusion in the dermis of leptin-overexpressing pigs but not in the control pigs. Original magnification, 100×. (c,d) Statistical analysis of the bleeding spots and the number of RBCs (*n* = 5). (e) Platelet and leukocyte number (*n* = 6). (f) CRP level examination. (g,h) Routine examination to analyze thrombin-associated factors (*n* = 4). (i) APA content evaluated by ELISA (*n* = 6). (j) The criterion for SLE characterization. *p < 0.05, *p < 0.01, ***p < 0.001, two-tailed Student *t* test for c,d,e,f,g,i. Data are presented as mean ± SEM. Numerical values are provided in S1 Data. APA, antiplatelet antibody; ARA, American Rheumatism Association; CRP, C-reactive protein; H&E, hematoxylin and eosin; RBC, red blood cell; SLE, systemic lupus erythematosus.

Additionally, at 6 months of age, the transgenic leptin pigs showed widespread subcutaneous bleeding spots, especially on the ears (Fig 1H) and backs, after strong exercise (Fig 2A). The bleeding spots progressed to erythema after two additional weeks (Fig 1I). Eventually, all transgenic pigs died at 7.5 ± 0.3 months age (Fig 1J) because of acute inflammation of the lungs (Fig 1K) and gastrointestinal tract (S1L Fig).

### Hematologic changes in transgenic leptin pigs

To identify the mechanisms underlying the observed subcutaneous bleeding in the transgenic leptin pigs, we performed hematoxylin and eosin (H&E) staining of the dermis to examine the histological changes that occurred. We found that there was erythrocyte leakage from the blood vessels in the dermis of transgenic leptin pigs (Fig 2B and 2C).

To further characterize the bleeding disorder observed, we measured whole red blood cells (RBCs) and found that there were fewer RBCs (5.45 ± 0.36 × 10^12^/L versus 8.05 ± 0.73 × 10^12^/L, *p* < 0.001; Fig 2D) and significantly fewer platelets (68.5 ± 27.9 × 10^9^/L versus 326 ± 20.7 × 10^9^/L, *p* < 0.001; Fig 2E) in the transgenic pigs compared to the control pigs, which may be the result of an RBC abnormality. Additionally, coagulation tests were performed to further diagnose the transgenic leptin pigs with leucopenia (Fig 2E). However, despite these observed abnormalities in the transgenic leptin pigs, they had a normal level of C-reactive protein (Fig 2F). Interestingly, there was no significant difference in the prothrombin times (PTs), international normalized ratios (INRs), and activated partial thromboplastin times (APTTs) between the transgenic leptin pigs and control pigs (S3 Fig). These results excluded a coagulation pathway as the cause of the subcutaneous bleeding in the transgenic leptin pigs. However, the transgenic leptin pigs had a lower level of fibrinogen protein (3.67 ± 0.16 g/L versus 4.44 ± 0.21 g/L, *p* < 0.05; Fig 2G) and consistently longer thrombin times (42.6 ± 3.7 s versus 20.5 ± 3.9 s, *p* < 0.05; Fig 2H) compared to the control pigs.

To identify whether the RBCs were diluted or there was a reduction in the number of RBCs contributing to the bleeding, we measured the average hemoglobin (HGB) content/concentration to determine the HGB volume in RBCs, as well as the RBC distribution width Coefficient of Variation (CV)/Standard Deviation (SD) in order to characterize the RBC morphologies. There was no significant difference in either of these markers between the transgenic leptin pigs and control pigs (S3 Fig). All together, these results confirmed that a dilution of RBCs was involved in the development of thrombocytopenia. To further explain the reduced platelet number, we performed H&E staining of the bone marrow in order to exclude leukemia as the cause of the platelet numbers. There were no indications of leukemia or other pathological changes (S4 Fig). Meanwhile, antiplatelet antibody (APA) levels were significantly higher in the transgenic leptin pigs than in control pigs (Fig 2I). Based on these results, the transgenic leptin pig model expressed a combination of markers and symptoms that fulfilled the 1999 American College of Rheumatology SLE classification criteria (Fig 2J).

### Impairment of liver and kidney function in transgenic leptin pigs

**Fig 3:**
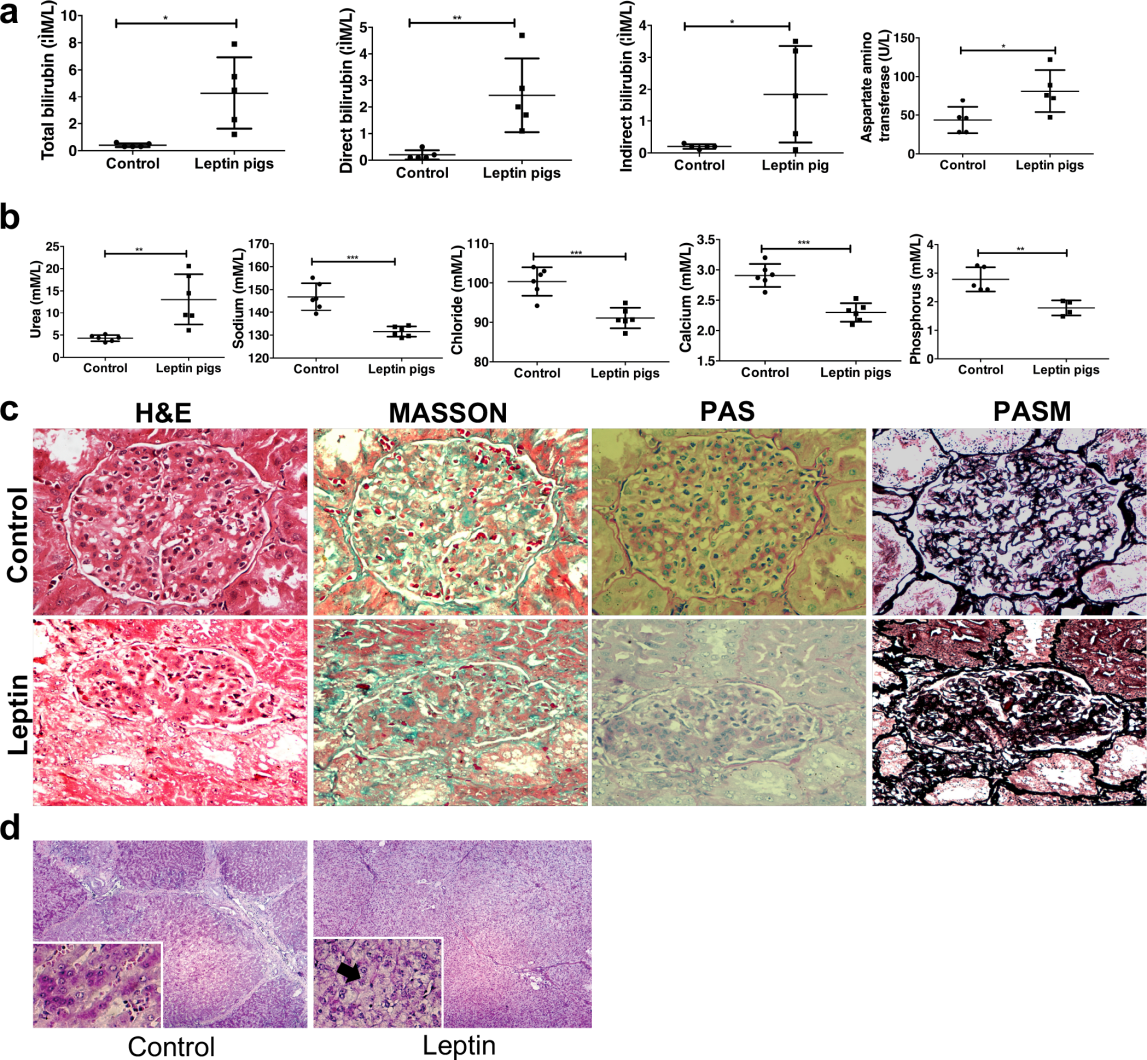
Pathological changes of renal or hepatic cells in transgenic leptin pigs. (a) Routine markers that reflect liver and kidney physiological functions. Aspartate amino transferase concentration was elevated in leptin-overexpressing pigs (*n* = 6 per group). (b) Examination of the levels of several ions in the peripheral blood of leptin-overexpressing pigs. Blood urea concentration was elevated in leptin-overexpressing pigs (*n* = 6 per group). (c) Serial sections of kidney tissues analyzed by H&E/Masson/PAS/PASM indicate the presence of renal injury in leptin-overexpressing pigs. Original magnification, 400×. (d) H&E staining indicates hepatocellular balloon changes in the hepatocytes of leptin-overexpressing pigs. Original magnification, 100×. *p < 0.05, *p < 0.01, ***p < 0.001, two-tailed Student *t* test for a,b. Data are presented as mean ± SEM. Numerical values are provided in S1 Data. H&E, hematoxylin and eosin; PAS, Periodic Acid–Schiff; PASM, Periodic Acid–Silver Methenamine.

Multiple organ injuries, especially involving the kidney, commonly occur in SLE patients. Thus, to further confirm our diagnosis of SLE in our transgenic leptin pigs, we performed serum biochemical analyses to assess the functions of the liver and kidney in the transgenic leptin pigs. Specifically, the bilirubin levels—including both direct and indirect bilirubin—and aspartate amino transferase (Fig 3A) and urea (Fig 3B) levels were significantly higher in the transgenic leptin pigs than in control pigs. In addition, the transgenic leptin pigs had reduced ionic concentrations of sodium, chloride, calcium, and phosphorus (Fig 3B) but not iron and magnesium (S5 Fig) when compared to the controls. These blood biochemistry results indicated that the transgenic leptin pigs had damaged liver and kidney functions.

To further confirm the injuries to the liver and kidney in the transgenic leptin pigs, histological analyses were performed on these organs, including H&E, Masson, Periodic Acid–Schiff (PAS), and Periodic Acid–Silver Methenamine (PASM). The kidneys of the transgenic leptin pigs exhibited a diffuse (>90%) glomerular involvement, including mesangial proliferation and glomerular basement membrane (GBM) thickening (Fig 3C). The livers of the transgenic leptin pigs had enlarged hepatic cell sizes compared to the controls as well as constricted hepatic sinusoids at low magnification. Significantly, at high magnification, the hepatocytes in the transgenic leptin pigs were characterized by hydropic degeneration and balloon-like changes in 70% of the hepatocytes, which was indicative of severe liver cell damage (Fig 3D). Interestingly, there were neither obvious pathological changes nor immunoglobulin G (IgG) deposition (S6 Fig) in the transgenic cloned pig and the cloned pig we described recently [22,23]. Together, these histological results further confirmed that the liver and kidney were injured in the transgenic leptin pigs (Fig 2J).

### Deposition of immunocomplexes detected in transgenic leptin pigs

**Fig 4:**
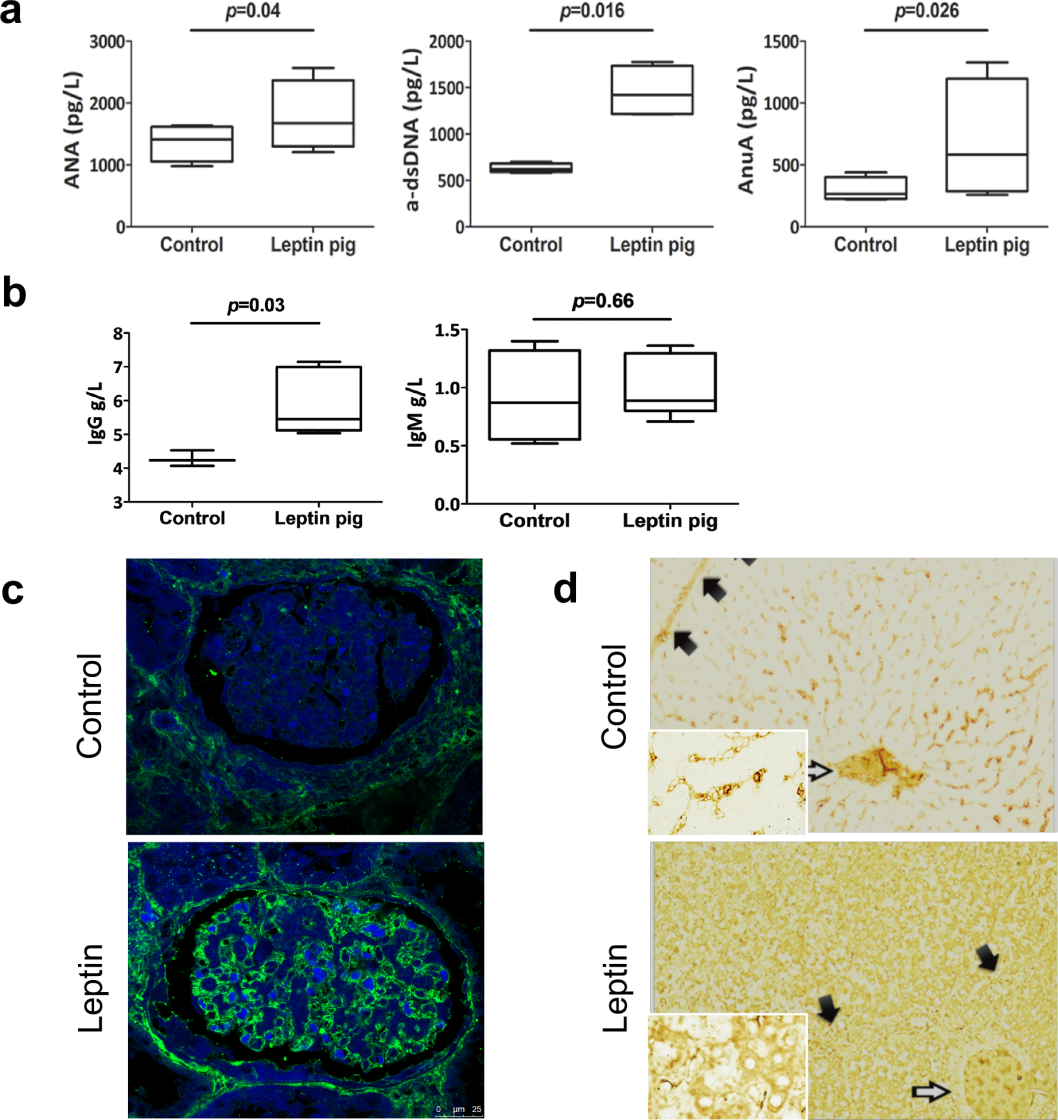
Autoantibody examination and Ig deposition detection for characterization of SLE. (a) ANA, α-dsDNA, and ANuA concentrations in serum were analyzed by ELISA (*n* = 6 per group). (b) The levels of IgG and IgM were detected (*n* = 6 per group). (c) Sections of the kidney (original magnification, 400×) and (d) liver (original magnification, 100×) were analyzed by immunofluorescence (IgG [green] deposition and DAPI [blue]) or IHC (IgG [brown]) to reveal the IgG deposition. *p* < 0.05 shows significance difference, two-tailed Student *t* test for a,b. Data are presented as mean ± SEM. Numerical values are provided in S1 Data. ANA, antinuclear antibody; ANuA, antinucleosome antibody; α-dsDNA, anti-dsDNA; dsDNA, double-stranded DNA antibody; Ig, immunoglobulin; IHC, immunohistochemistry; SLE, systemic lupus erythematosus.

In SLE patients, pathological changes in organs and tissues are often accompanied by depositions of immunocomplexes [24] and serum immunologic changes characterized by the appearance or elevation of various autoantibodies. The peripheral blood of the transgenic leptin pigs did in fact contain autoantibodies, including anti-antinuclear antibody (ANA) antibodies, anti-double-stranded–DNA antibodies (dsDNAs), and anti-antinucleosome antibodies (ANuAs). The levels of anti-dsDNA, anti-ANuA, and anti-ANA antibodies were all significantly elevated compared to the control pigs (Fig 4A). In the transgenic leptin pigs, there was no significant change in the level of IgM compared to the controls; however, IgA was undetectable (Fig 4B). To further confirm our diagnosis of SLE in the transgenic leptin pigs, immunoglobulins (Igs) and autoantibodies were measured using immunofluorescence/immunohistochemistry (IF/IHC) staining. We found strong evidence of IgG deposition in the global glomeruli (Fig 4C), as well as in the hepatocytes and hepatic sinusoidal capillary endothelium (Fig 4D), in the transgenic leptin pigs. Infiltration of CD4+ T lymphocytes was also detected around renal vessels (S7 Fig). Interestingly, the deposition of IgG was only detectable in the kidney and liver but not in the pancreas, brain, intestine, or lung (S8 Fig).

### Repression of Tregs in transgenic leptin pigs

**Fig 5:**
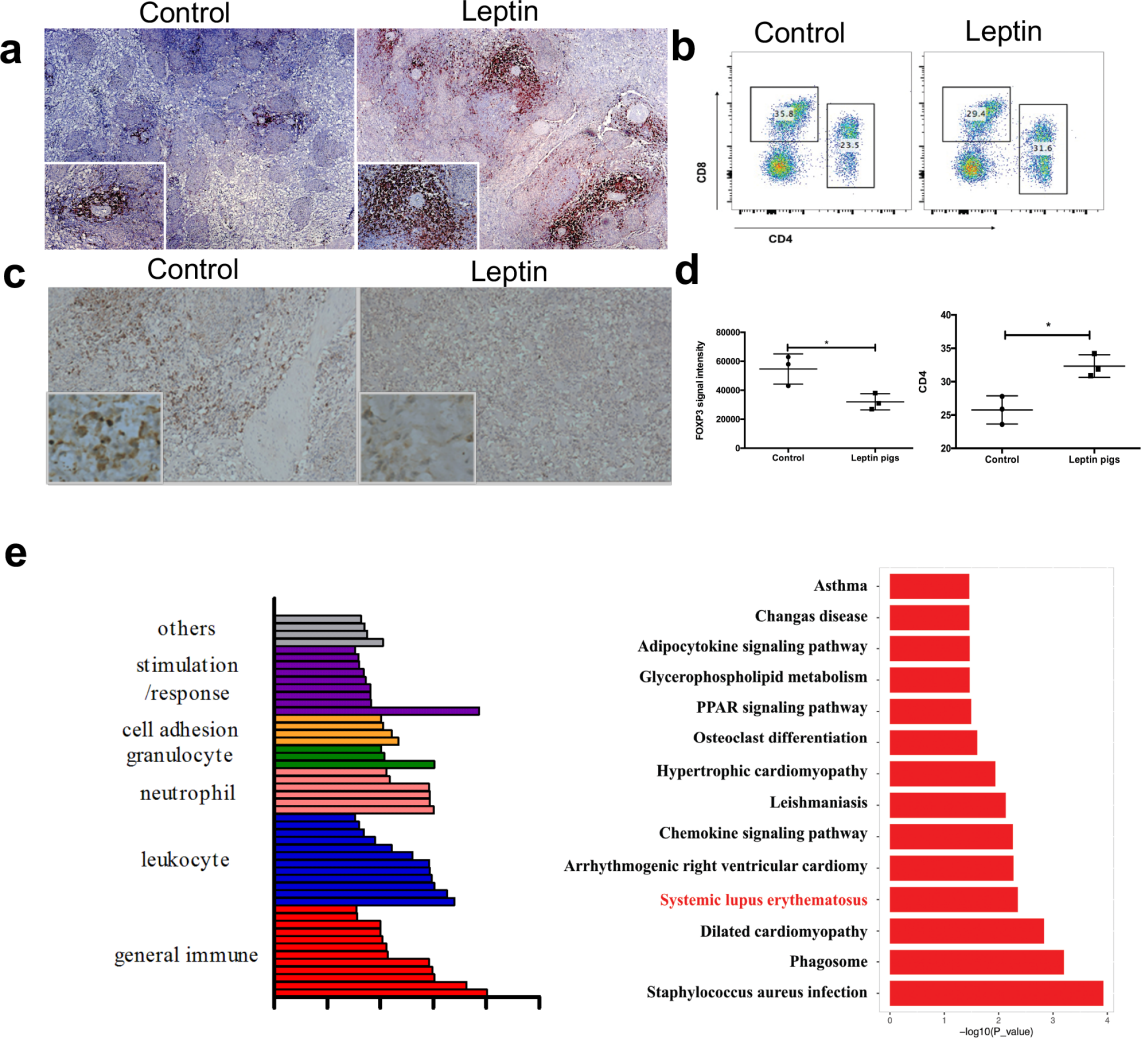
Effects of leptin on the immune system. Sections of spleen tissues from both control and transgenic leptin pigs were probed with (a) CD4 and (b) FOXP3 antibodies. (c) The ratio of CD4+/CD8+ cells in PBMCs was detected by flow cytometric analysis. (d) Five random scopes were captured for statistical analysis. The mean level of Foxp3 to CD4 signal intensity ratio and standard error bars are presented (*n* = 3 per group). (e) RNA-seq was performed on PBMCs to determine the transcriptome change in the transgenic leptin pigs (*n* = 3 per group). Summary of gene-significant differences in KEGG pathways. Histogram of the GO classifications of gene-significant differences. *p < 0.05, two-tailed Student *t* test for d,e. Data are presented as mean ± SEM. Numerical values are provided in S1 Data. GO, gene ontology; KEGG, Kyoto Encyclopedia of Genes and Genomes; PBMC, peripheral blood mononuclear cell; PPAR, Peroxisome proliferator-activated receptors; RNA-seq, RNA sequencing.

To better understand the global immunologic changes occurring in the transgenic leptin pigs, we profiled the circulating immune cells using flow cytometry. Interestingly, leptin affects several types of immune cells, including CD4+ T lymphocytes and CD21+ B cells at the pre-disease stage, but it has minimal effects on NK cells. (Fig 5A and 5B, S9 Fig). The largest differences in immune cells between the transgenic leptin pigs and the controls were observed in the Tregs. To explore how effector T cells were elevated, Tregs were measured in the spleen. We found that the level of Tregs was suppressed in the spleens of the transgenic leptin pigs (Fig 5C and 5D). To identify how Tregs were suppressed in the transgenic pigs, we used leptin to stimulate T cells and found that fewer naïve T cells differentiated into Tregs compared to the control pigs (S10 Fig). RNA sequencing (RNA-seq) was performed to detect the transcriptomic changes in peripheral blood mononuclear cells (PBMCs) in the transgenic leptin pigs. With 2-fold change and *p* < 0.05 threshold, we found that 84 transcripts were up-regulated and 124 transcripts were down-regulated in the transgenic leptin pigs. Interestingly, leptin pigs showed reduced expression of members of the C-C/C-X-C motif chemokine ligand family, including C-C motif chemokine ligand 4 (CCL4), C-X-C motif chemokine ligand 2 (CXCL2), and C-C motif chemokine ligand 3 like 1. Next, ontological analyses were performed for those 208 genes that showed a difference in expression. Interestingly, most of these genes are related to immune function, including general immune function, immune leukocytes, neutrophils, and granulocytes. (Fig 5E, S2 Data). Additionally, the JAK/STAT pathway, which is considered to be a major downstream pathway of leptin, was the leading hyperactivated pathway in our screen. Western blots were performed to verify the activation of phospho-ERK, JAK2, and STAT3 (S11 Fig).

### Inhibitory immune therapy elevates APA levels and relieves subcutaneous bleeding

**Fig 6:**
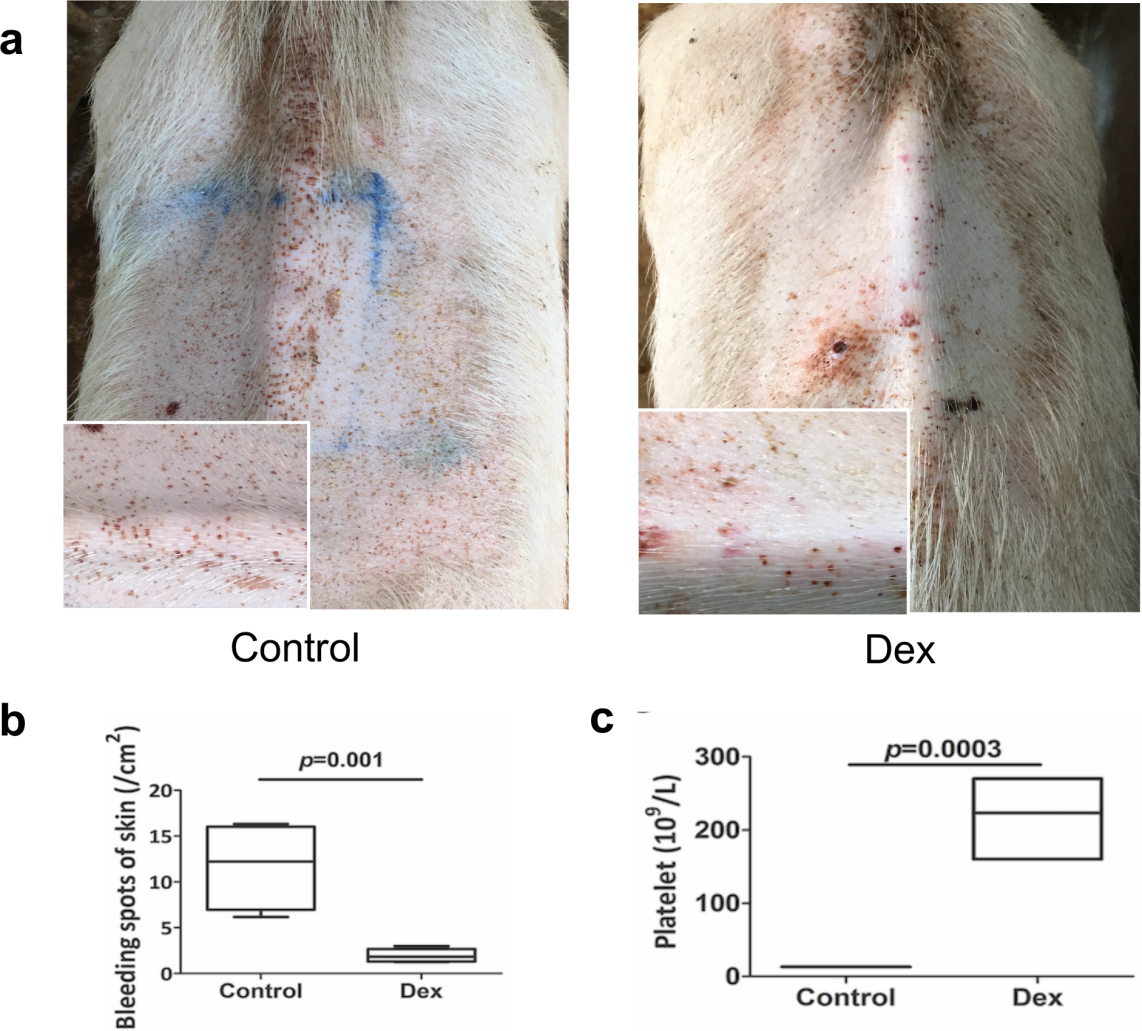
Inhibitory immune therapy rescues the subcutaneous bleeding phenotype. (a) An image of leptin-overexpressing pigs with or without Dex treatment. (b) Statistical analysis of the bleeding spots (control *n* = 1, Dex *n* = 3). (c) Platelet content in both groups of pigs (control *n* = 1, Dex *n* = 3). *p* < 0.05 shows significance difference, two-tailed Student *t* test for b,c. Data are presented as mean ± SEM. Numerical values are provided in S1 Data. Dex, dexamethasone.

In SLE, glucocorticoid treatment is often considered to be an effective and necessary therapeutic strategy. Therefore, we performed experimental glucocorticoid therapies in our transgenic leptin pigs. Three transgenic leptin pigs were intravenously injected with dexamethasone (10 mg/pig/day) via the ear vein continuously for 2 weeks. A healthy control pig was examined for subcutaneous bleeding, body weight, liver and kidney functions, and blood biochemistry. We detected a decrease in the symptoms of SLE after dexamethasone treatment in the transgenic leptin pigs. Remarkably, the number of bleeding spots on the skin was significantly reduced in the dexamethasone group compared with the control group (Fig 6A and 6B), accompanied by an increase in the platelet number (Fig 6C). Additionally, the transgenic leptin pigs exhibited better overall general conditions and improvement to liver and kidney dysfunctions.

## Discussion

SLE is an autoimmune disease characterized by various autoantibodies that affect multiple organs and systems. Leptin is also frequently elevated in SLE patients, and previous SNP analyses of leptin and the leptin receptor (LR) have revealed that there is an association between leptin/LR mutations and SLE [25]. Consistent with human data, our leptin transgenic pig model developed a number of pathophysiological changes with multiorgan injuries resembling SLE. Our work not only corroborates the association between leptin with SLE-like symptoms observed in human patients but more importantly implies a crucial role for leptin in promoting the development of SLE. Together, our data demonstrate that our transgenic leptin pig may be used as a novel porcine SLE model.

A series of clinical manifestations and immunological abnormalities characteristic of SLE were diagnosed in our transgenic leptin pigs. Three of those are consistent with the 1999 criteria for diagnosing SLE proposed by the American Rheumatism Association and involve both clinical and immunological aspects [20]. These symptoms include 1) a decrease in leukocyte and platelet count in the whole blood; 2) histopathological changes in liver, kidney, and other organs resembling the multiorgan injuries elicited by SLE; and 3) a marked increase of ANAs and anti-dsDNAs. In addition, clinical and biochemical characteristics of our transgenic leptin pigs also corresponded with the 2009 Systemic Lupus International Collaborating Clinics (SLICC) revised criteria [26], including the previously mentioned abnormalities and renal pathological changes.

It is important to note that hematological systems are one of the most frequently impacted areas in SLE patients. As a proinflammatory adipokine, leptin affects both adaptive and innate immunity [27]. Interestingly, we found that leptin overexpression in pigs induced considerable alterations in the immune system and resulted in the presence of various autoantibodies in the whole blood and immune complex deposition in organs such as the kidney. Further research showed a significant decrease in the percentage of Tregs, which was consistent with changes observed in patients with active SLE. The potential role of leptin in SLE that we demonstrated here is also consistent with previous reports showing that a genetic deficiency of leptin protected mice from SLE, specifically reducing the production of autoantibodies and preventing renal disease [28]. Particularly, the gene expression profiling of our transgenic leptin pigs showed changes consistent with the typical alterations to proinflammatory pathways observed in human SLE patients. Altogether, our data show that leptin overexpression in our transgenic leptin pigs promotes autoimmune phenotypes resembling human SLE macroscopically, histologically, and molecularly.

Obesity is associated with many human diseases, including autoimmune diseases, metabolic syndrome, and inflammation [29–31]. Our work provides compelling evidence indicating that an increased leptin level plays a crucial role in the development of obesity-associated SLE.

Murine models are useful tools for researching lupus pathogenesis, and they are well documented as mimicking human SLE at the molecular and cellular level. However, most of the models only partially resemble human SLE [27] and are used to study certain aspects of lupus pathogenesis [32–35]. A number of SLE-prone mouse models have been developed for use in SLE studies [32,33,36,37]. The SLE-prone NZM2410 mouse model is highly penetrant, with early-onset lupus nephritis in both males and females [32,33]. The MRL/Mp mouse model presented some SLE-like symptoms, including autoimmune antibody production in the peripheral blood [36,37]. However, these mouse models are unable to fully demonstrate all the features of human SLE patients, especially when looking at the mechanisms underlying the disease progression. In a spontaneous animal model of SLE, the administration of leptin promoted acceleration of the disease, whereas functionally reducing leptin protected animals from the development of autoimmunity [28]. However, lupus can be extremely complex and exhibit high clinical heterogeneity, which was not fully demonstrated in the leptin-overexpressing mice.

C-X-C motif ligands/C-C motif chemokine ligands have been implicated in the pathogenesis of diseases characterized by monocytic infiltrates, like psoriasis, rheumatoid arthritis, and atherosclerosis. We found CCL4 is the most down-regulated gene in PBMCs from leptin pigs. CCL4 has not yet been linked with SLE. It has been shown to play a critical role in Treg function. Tregs deficient for expression of CCL4 were impaired in their ability to suppress experimental autoimmune encephalomyelitis or islet allograft rejection in murine models [38]. This is consistent with the reduction of Treg portion in leptin pigs. Besides JAK2/STAT3 and other pathways we detected, “metabolic pressure” has been reported to mediate the leptin signal to immune response. Imbalanced glucose, amino acids, and lipids metabolism, induced by an elevated leptin signal, will compromise Th1/Th17/Treg homeostasis [39].

Pigs have similar body sizes, anatomical structures, organ physiology, and life spans to humans [15]. In this study, the leptin-overexpressing pigs exhibited remarkable resemblance to humans with SLE in multiple aspects of the disease. Similar to the ways in which a pig model has been influential in studying cystic fibrosis [18], we believe that the leptin-overexpressing transgenic pig is a promising preclinical SLE model that could be influential in performing translational research to further understand this disease and determine beneficial treatment strategies.

## Materials and methods

### Ethics statement

The miniature pigs used in our study were regularly maintained at the Animal Center of Yunnan Agricultural University. All experiments involving pigs for the humane endpoints practiced in our study were approved by the Institutional Animal Care and Use Committee of Yunnan Agricultural University (permission code: YAUACUC01; date of publication: 10 July 2013).

### Vector construction

The pleptin ORF was amplified from a cDNA library prepared from mRNA purified from a fetal pig brain. After purification, the PCR products were inserted into pMD18-T using the T/A cloning method. Leptin-positive clones were selected by PCR with pleptin RT primers. The plasmid pMD18-T was digested by SalI and EcoRI and inserted into pIRES2-AcGFP. Sequencing was performed to confirm the insert.

### PCR and semiquantitative PCR

The sequences and corresponding PCR information for the primers used in this study are listed in S1 Table. Premix Ex Taq polymerase (Takara D332) was used for PCRs. Trizol (Invitrogen; Carlsbad, CA, USA) was used for RNA extraction and purification. The PrimeScriptTM RT kit (Takara RR047A) was used to perform the reverse transcription reactions. A T100 Thermal Cycler (Bio-Rad; Hercules, CA, USA) was used for PCR amplification. DNA electrophoresis was performed using a Mini-Sub cell GT system and a Gel imaging system (Bio-Rad). All oligonucleotides used in this study are listed in S1 Table.

### Animals

Pig fibroblast cells were isolated from 30-day-old York pig embryos using a previously described method [22]. The cells (1 × 10^6^) were washed twice after trypsin digestion and mixed with 6 μg/ml plasmid DNA before being transferred into a cuvette with a 0.4 cm gap. Electroporation was performed at 250 v for 25 ms. Neomycin (500 μg/mL) was added into the media for 2 weeks to select the stably transfected cell line. SCNT was performed using a standard procedure [22,23]. The activated oocytes containing the nucleus from the leptin fibroblast cells were transplanted into the uteri of surrogate sows. On average, approximately 400 developing embryos were transplanted into one surrogate sow. A total of six surrogate sows were used in this study, and five of them became pregnant. The newborn pigs were removed by cesarean section. A total of 13 pigs survived. All pigs were raised to sexual maturity at 6 months old. Standard procedures for animal husbandry were used throughout the study. Animal operations were performed under general anesthesia after Propofol ear injection (dose = 2.5 mg/kg). Blood was collected from the precaval vein and used in physiological examinations. Scoring with the Mouse Interventional Scoring System (MISS) was a surrogate for death as an endpoint and was tested concurrent to main investigative aims. The experimental endpoint was followed according to the MISS [40]. We gave each clinical feature—including appearance, respiratory rate, general behavior, provoked behavior, and weight loss—a numerical value and scored them. Each criterion contains 0 to 12 points. Pigs were euthanized by Propofol ear injection when the score was equal to or higher than 12.

### Subcutaneous bleeding spots analysis

Images of three random areas from the back of each pig were taken. The number of subcutaneous bleeding spots was counted and analyzed by GraphPad software.

### OGTT

Pigs were fasted overnight. Each pig received a total of 100 grams of glucose dissolved in water by injection into the pigs’ throats. The blood was collected from an ear vein at the indicated time points.

### Biopsy, H&E staining, and IHC

Tissues for biopsy were removed using a puncture needle under the direction of a Doppler ultrasonic apparatus. Paraffin-embedded tissue sections and H&E staining were performed using standard processes as previously described [39]. IHC was performed as previously described [41]. Commercially available antibodies were purchased from Abcam (New York, NY, USA) or Santa Cruz Biotechnology (Santa Cruz, CA, USA; Leptin, Ab16227, 1:200 dilution; CD4, 203034, 1:200 dilution; pSTAT3, SC-8001, 1:100 dilution). The corresponding IgG and secondary antibodies were purchased from Santa Cruz. For the liver/kidney injury analysis based on H&E, a total of 200 renal capsules or 500 hepatocellular cells from multiple sections were counted. The signal analysis for IHC was performed using Image Pro Plus 6.0 software.

### APA, α-dsDNA, ANA, and AnuA ELISA

Serum from both groups of pigs was used in the APA ELISA. APA IgG (SBJ-0805G), IgM (SBJ-0805M), IgA (SBJ-0805A), α-dsDNA (SBJ-201507), ANA(SBJ-201508), and AnuA (SBJ-201509) ELISA kits were purchased from Nanjing SENBEIJIA Company. Standard procedures were performed according to the manufacturers’ instructions.

### Flow cytometric analysis

For surface staining, the following fluorochrome-labeled mAbs from BD Bioscience were used: anti-CD3 (FITC-559582) and anti-CD4 (PE-561473), CD8 (APC-561475), CD21 (PE-Cy7-561374), CD16 (551395), and from SouthernBiotech, CD56 (Pacific Blue-9456-27). Flow cytometry was acquired on a FACSCalibur system (BD Biosciences) using Cell-Quest (BD Biosciences) software, and data were analyzed using Flowjo software (Tree Star).

### RNA-seq and data analysis

Sequencing libraries were constructed according to the protocol for the Illumina TruSeq Sample preparation kit. Sequencing was performed on the Illumina HiSeq 2500 sequencer. Library construction and sequencing were performed at the Genergy Biotech Co., Ltd. (Shanghai, China). Paired-end RNA-seq reads with 101 bp at each end were aligned to the ENSEMBL version 75 transcriptome database using the Bowtie program [42]. The gene-expression levels were quantified using RSEM software [43]. Differential expression analysis was conducted using maSigPro [44]. The significant genes were selected using an FDR cutoff value of 0.05. The Go-process program in Metacore software (https://portal.genego.com/) was used to analyze the RNA-seq data. The RNA-seq dataset has been deposited in the database at NCBI-GEO (https://www.ncbi.nlm.nih.gov/geo/query/acc.cgi?acc=GSE116701).

### Medical apparatus in routine examination

A SysMex xs-800i was used for routine blood tests. An Acl Top 700 was used for the thrombin test. A Beckman Coulter Image 800 was used for the immunological tests. An Olympus AU5400 was used for the hepatic and renal physiological functional tests. A Roche Elecsys 2010 was used for the insulin tests. All routine examinations were repeated twice from different samples collected separately and were combined for statistical analysis.

### Medicine

The following drugs were used in experiments and given via injection: Propofol solution (Guangdong Jiabo Pharmaceuticals Company), sodium chloride solution (Zhejiang Medicine Company), and dexamethasone sodium phosphate solution (Beijing Shuanghe Medicine Company).

### Western blotting

PBMCs were lysed in radioimmunoprecipitation assay buffer supplemented with protease and phosphatase inhibitor cocktails (Thermo Scientific, #78440). Mouse anti-actin Ab (1:5,000; 3700S, Cell Signaling Technology), rabbit anti-p-stat3 Ab (1:1,000; sc-8001, Santa Cruz), rabbit anti-p-erk1/2 Ab (sc-13073, Santa Cruz), and rabbit anti-jak2 Ab (sc-278, Santa Cruz) were used. Data were analyzed using Image Lab software (Bio-Rad). Images have been cropped for presentation.

### Statistics

Statistical analysis and diagram illustrations were performed using Graphpad Prism 6 software. Experimental differences were evaluated using the unpaired two-tailed *t* test, one-way analysis. Data are presented as mean ± SEM.

## Supporting information

S1 Protocol Construction of leptin-overexpression pigs. (DOCX)

S1 Fig IHC results verify leptin expression in the pancreas (upper panel, 100× magnification) and pSTAT3 elevation in the spleen (lower panel, 100× magnification). IHC, immunohistochemistry.
(TIF)

S2 Fig Sections of spleen tissues. (a) The sections of spleen tissues from both control and transgenic leptin pigs were probed with ERK2, JAK2, STAT3, and STAT5 antibodies. (b) Nine scopes were randomly captured for statistical analysis. (c) Serum insulin levels in pigs. The mean level of signal intensity and standard error bars are presented (*p < 0.05, **p < 0.01).
(TIF)

S3 Fig Coagulation tests and RBC and HGB examinations. HGB, hemoglobin; RBC, red blood cell.
(TIF)

S4 Fig H&E staining results from section of bone marrow from transgenic leptin pigs. H&E, hematoxylin and eosin.
(TIF)

S5 Fig Iron and magnesium levels. (TIF)

S6 Fig Sections from the kidneys of the transgenic leptin pigs, Banna miniature inbred pigs, and GTKO/hCD55/hCD59 pigs were analyzed by H&E and IHC to assess the IgG deposition. The SCNT technique was used to generate the leptin pigs. H&E, hematoxylin and eosin; Ig, immunoglobulin; IHC, immunohistochemistry; SCNT, somatic cell nuclear transfer.
(TIFF)

S7 Fig IHC staining of CD4+ T lymphocytes in kidney of transgenic leptin pig. IHC, immunohistochemistry.
(TIF)

S8 Fig Sections of multiple tissues were analyzed by H&E and IHC to assess the IgG deposition. H&E, hematoxylin and eosin; Ig, immunoglobulin; IHC, immunohistochemistry.
(TIF)

S9 Fig B and NK cells in PBMCs were detected by flow cytometric analysis. NK, natural killer; PBMC, peripheral blood mononuclear cell.
(TIF)

S10 Fig Leptin inhibited the conversion of mice CD4+CD25− T cells into Tregs. Magnetic-bead–sorted CD4+CD25− T cells were incubated with 2.5 μg/mL anti-CD3/CD28 Ab for 3 d. The culture media for the Tregs contained added TGF-β (0.1 ng/μL), with or without leptin (100 ng/mL). Cells were analyzed by FACS 3 d later. FACS, fluorescence-activated cell sorting; Treg, regulatory T cell.
(TIF)

S11 Fig Phospho-ERK, JAK2, and STAT3 were analyzed by western blot. 10% of the lysate was used to detect total levels of the respective proteins.
(TIF)

S1 Table Oligonucleotides. (DOCX)

S1 Data The raw data of numerical values. (XLSX)

S2 Data Differential expression genes. (XLS)

## Abbreviations

α-dsDNA: anti-dsDNA
ANA: antinuclear antibody
ANuA: antinucleosome antibody
APA: antiplatelet antibody
APTT: activated partial thromboplastin time
ARA: American Rheumatism Association
CCL4: C-C motif chemokine ligand 4
CRP: C-reactive protein
CXCL2: C-X-C motif chemokine ligand 2
CV: Coefficient of Variation
dsDNA: double-stranded DNA antibodies
GBM: glomerular basement membrane
GFP: green fluorescent protein
GO: gene ontology
H&E: hematoxylin and eosin
HGB: hemoglobin
Ig: immunoglobulin
IHC: immunohistochemistry
IHF/C: immunofluorescence/immunohistochemistry
IL2RG: interleukin-2 receptor subunit-gamma
INR: international normalized ratio
KEGG: Kyoto Encyclopedia of Genes and Genomes
LR: leptin receptor
MISS: Mouse Interventional Scoring System
NK: natural killer
OGTT: oral glucose tolerance test
ORF: open reading frame
PAS: Periodic Acid–Schiff
PASM: Periodic Acid–Silver Methenamine
PBMC: peripheral blood mononuclear cell
pleptin: pig leptin
PT: prothrombin time
RBC: red blood cell
RNA-seq: RNA sequencing
SCID: Severe Combined Immunodeficiency
SCNT: somatic cell nuclear transfer
SD: Standard Deviation
SLE: systemic lupus erythematosus
SLICC: Systemic Lupus International Collaborating Clinics
T/A cloning: Original TA Cloning
Th: helper T
Treg: regulatory T cell

## References

[1] Lisnevskaia L, Murphy G, Isenberg D. Systemic lupus erythematosus. Lancet. 2014;384(9957):1878–88. 10.1016/S0140-6736(14)60128-8 24881804

[2] Maroñas JL, Gutiérrez GC, Muley AR, Guerra TA. [Systemic lupus erythematosus]. Lancet. 2014;384(9957):1878–88. 10.1016/S0140-6736(14)60128-8 24881804

[3] Barnett R. Systemic lupus erythematosus. The Lancet. 2016;387(10029):1711 10.1016/s0140-6736(16)30266-5 27116268

[4] Koutsokeras T, Healy T. Systemic lupus erythematosus and lupus nephritis. Nature Reviews Drug Discovery. 2014;13(3):173 10.1038/nrd4227 24525782

[5] Lewis M, Vyse S, Shields A, Boeltz S, Gordon P, Spector T, et al Effect of UBE2L3 genotype on regulation of the linear ubiquitin chain assembly complex in systemic lupus erythematosus. Lancet. 2015;385 Suppl 1:S9. 10.1016/S0140-6736(15)60324-5 26312912

[6] Wilbe M, Jokinen P, Truvé K, Seppala EH, Karlsson EK, Biagi T, et al Genome-wide association mapping identifies multiple loci for a canine SLE-related disease complex. Nature Genetics. 2010;42(3):250 10.1038/ng.525 20101241

[7] Han JW, Zheng HF, Cui Y, Sun LD, Ye DQ, Hu Z, et al Genome-wide association study in a Chinese Han population identifies nine new susceptibility loci for systemic lupus erythematosus. Nature Genetics. 2009;41(11):1234–7. 10.1038/ng.472 19838193

[8] Wahren-Herlenius M, Dörner T. Immunopathogenic mechanisms of systemic autoimmune disease. Lancet. 2013;382(9894):819–31. 10.1016/S0140-6736(13)60954-X 23993191

[9] Fernández-Riejos P, Najib S, Santos-Alvarez J, Martín-Romero C, Pérez-Pérez A, González-Yanes C, et al Role of leptin in the activation of immune cells. Mediators of inflammation. 2010;2010. 10.1155/2010/568343 PMC2846344 20368778

[10] Mestas J, Hughes CC. Of mice and not men: differences between mouse and human immunology. The Journal of Immunology. 2004;172(5):2731–8. 14978070 10.4049/jimmunol.172.5.2731

[11] Procaccini C, Jirillo E, Matarese G. Leptin as an immunomodulator. Molecular Aspects of Medicine. 2012;33(1):35–45. 10.1016/j.mam.2011.10.012 22040697

[12] De-Rosa V, Procaccini C, G, Pirozzi G, Fontana S, Zappacosta S, La-Cava A, et al A Key Role of Leptin in the Control of Regulatory T Cell Proliferation. Immunity. 2007;26(2):143–5. 10.1016/j.immuni.2007.02.002 17307705

[13] Lord GM, Matarese G, Howard JK, Baker RJ, Bloom SR, Lechler RI. Leptin modulates the T-cell immune response and reverses starvation-induced immunosuppression. Nature. 1998;394(6696):897–901. 10.1038/29795 9732873

[14] Abella V, Scotece M, Conde J, Pino J, Gonzalezgay MA, Gómezreino JJ, et al Leptin in the interplay of inflammation, metabolism and immune system disorders. Nature Reviews Rheumatology. 2017;13(2):100–9. 10.1038/nrrheum.2016.209 28053336

[15] Meurens F, Summerfield A, Nauwynck H, Saif L, Gerdts V. The pig: a model for human infectious diseases. Trends in Microbiology. 2012;20(1):50 10.1016/j.tim.2011.11.002 22153753 PMC7173122

[16] Keiser NW, Engelhardt JF. New animal models of cystic fibrosis: what are they teaching us? Current Opinion in Pulmonary Medicine. 2011;17(6):478–83. 10.1097/MCP.0b013e32834b14c9 21857224 PMC3596000

[17] Suzuki S, Iwamoto M, Saito Y, Fuchimoto D, Sembon S, Suzuki M, et al Il2rg gene-targeted severe combined immunodeficiency pigs. Cell Stem Cell. 2012;10(6):753–8. 10.1016/j.stem.2012.04.021 22704516

[18] Hoegger MJ, Fischer AJ, McMenimen JD, Ostedgaard LS, Tucker AJ, Awadalla MA, et al Impaired mucus detachment disrupts mucociliary transport in a piglet model of cystic fibrosis. Science. 2014;345(6198):818–22. 10.1126/science.1255825 ; PubMed Central PMCID: PMCPMC4346163. 25124441 PMC4346163

[19] Lourenço EV, Liu A, Matarese G, La CA. Leptin promotes systemic lupus erythematosus by increasing autoantibody production and inhibiting immune regulation. Proceedings of the National Academy of Sciences of the United States of America. 2016;113(38):10637 10.1073/pnas.1607101113 27588900 PMC5035847

[20] nomenclature TACoR, syndromes cdfnl, Arthritis, rheumatism. The American College of Rheumatology nomenclature and case definitions for neuropsychiatric lupus syndromes. Arthritis & Rheumatology. 1999;42(4):599–608. 10.1002/1529-0131(199904)42:4<599::AID-ANR2>3.0.CO;2-F 10211873

[21] Simonds S, Pryor J, Ravussin E, Greenway F, Dileone R, Allen A, et al Leptin Mediates the Increase in Blood Pressure Associated with Obesity. Cell. 2014;159(6):1404–16. 10.1016/j.cell.2014.10.058 25480301 PMC4259491

[22] Wei H, Qing Y, Pan W, Zhao H, Li H, Cheng W, et al Comparison of the efficiency of Banna miniature inbred pig somatic cell nuclear transfer among different donor cells. PLoS ONE. 2013;8(2):e57728 10.1371/journal.pone.0057728 23469059 PMC3585185

[23] Liu F, Liu J, Yuan Z, Qing Y, Li H, Xu K, et al Generation of GTKO Diannan miniature pig expressing human complementary regulator proteins hCD55 and hCD59 via T2A peptide-based bicistronic vectors and SCNT. Molecular Biotechnology. 2018; 60(8):550–562. 10.1007/s12033-018-0091-6 29916131

[24] Budhai L, Oh K, Davidson A. An in vitro assay for detection of glomerular binding IgG autoantibodies in patients with systemic lupus erythematosus. Journal of Clinical Investigation. 1996;98(7):1585–93. 10.1172/JCI118952 8833907 PMC507591

[25] Zhao J, Wu H, Langefeld CD, Kaufman KM, Kelly JA, Bae SC, et al Genetic associations of leptin-related polymorphisms with systemic lupus erythematosus. Clinical Immunology. 2015;161(2):157–62. 10.1016/j.clim.2015.09.007 26385092 PMC4658308

[26] Petri M, Orbai AM, Alarcon GS, Gordon C, Merrill JT, Fortin PR, et al Derivation and validation of the Systemic Lupus International Collaborating Clinics classification criteria for systemic lupus erythematosus. Arthritis Rheum. 2012;64(8):2677–86. 10.1002/art.34473 ; PubMed Central PMCID: PMCPMC3409311. 22553077 PMC3409311

[27] Abella V, Scotece M, Conde J, Pino J, Gonzalezgay MA, Gómezreino JJ, et al Leptin in the interplay of inflammation, metabolism and immune system disorders. Nature Reviews Rheumatology. 2017;13:100–109. 10.1038/nrrheum.2016.209 28053336

[28] Lourenco EV, Liu A, Matarese G, La Cava A. Leptin promotes systemic lupus erythematosus by increasing autoantibody production and inhibiting immune regulation. Proc Natl Acad Sci U S A. 2016;113(38):10637–42. Epub 2016/09/03. 10.1073/pnas.1607101113 ; PubMed Central PMCID: PMCPMC5035847. 27588900 PMC5035847

[29] Versini M, Jeandel PY, Rosenthal E, Shoenfeld Y. Obesity in autoimmune diseases: Not a passive bystander. Autoimmunity Reviews. 2014;13(9):981 10.1016/j.autrev.2014.07.001 25092612

[30] KM F, BI G, DF W. Cause-specific excess deaths associated with underweight,overweight,and obesity. Journal of the American Medical Association. 2007;298(17):2028–37. 10.1001/jama.298.17.2028 17986696

[31] Börgeson E, Johnson AF, Lee YS, Till A, Syed GH, Ali-Shah ST, et al Lipoxin A 4 Attenuates Obesity-Induced Adipose Inflammation and Associated Liver and Kidney Disease. Cell Metabolism. 2015;22(1):1550–4131. 10.1016/j.cmet.2015.05.003 PMC4584026 26052006

[32] Anderson CC, Cairns E, Rudofsky UH, Sinclair NR. Defective antigen-receptor-mediated regulation of immunoglobulin production in B cells from autoimmune strains of mice. Cellular Immunology. 1995;164(1):141–9. 10.1006/cimm.1995.1153 7634346

[33] Morel L, Yu Y, Blenman KR, Caldwell RA, Wakeland EK. Production of congenic mouse strains carrying genomic intervals containing SLE-susceptibility genes derived from the SLE-prone NZM2410 strain. Mammalian Genome. 1996;7(5):335–9. 8661718 10.1007/s003359900098

[34] Sang A, Yin Y, Zheng YY, Morel L. Animal models of molecular pathology systemic lupus erythematosus. Prog Mol Biol Transl Sci. 2012;105(105):321–70. 22137436 10.1016/B978-0-12-394596-9.00010-X

[35] Mizui M, Tsokos GC. Systemic Lupus Erythematosus, Animal Models In: Mackay IR, Rose NR, Diamond B, Davidson A, editors. Encyclopedia of Medical Immunology. New York: Springer; 2014 p. 1134–41.

[36] Steinberg AD, Roths JB, Murphy ED, Steinberg RT, Raveche ES. Effects of thymectomy or androgen administration upon the autoimmune disease of MRL/Mp-lpr/lpr mice. Journal of Immunology. 1980;125(2):871–3. 7391583

[37] Lewis DE, Giorgi, Amp JV, Warner NL. Flow cytometry analysis of T cells and continuous T-cell lines from autoimmune MRL/l mice. Nature. 1981;289(5795):298–300. 6969857 10.1038/289298a0

[38] Patterson SJ, Pesenacker AM, Wang AY, Gillies J, Mojibian M, Morishita K, et al T regulatory cell chemokine production mediates pathogenic T cell attraction and suppression. Journal of Clinical Investigation. 2016;126(3):1039 10.1172/JCI83987 26854929 PMC4767359

[39] De VR, La AC, Matarese G. Metabolic pressure and the breach of immunological self-tolerance. Nature Immunology. 2017;18(11):1190–6. 10.1038/ni.3851 29044230

[40] Koch A, Gulani J, King G, Hieber K, Chappell M, Ossetrova N. Establishment of Early Endpoints in Mouse Total-Body Irradiation Model. PLoS ONE. 2016;11(8):e0161079 10.1371/journal.pone.0161079 27579862 PMC5007026

[41] Zeng W, Su J, Wu L, Yang D, Long T, Li D, et al CD147 promotes melanoma progression through hypoxia-induced MMP2 activation. Current Molecular Medicine. 2014;14(1):163–73. 24090196 10.2174/15665240113136660077

[42] Langmead B, Trapnell C, Pop M, Salzberg SL. Ultrafast and memory-efficient alignment of short DNA sequences to the human genome. Genome Biology. 2009;10(3):R25 10.1186/gb-2009-10-3-r25 19261174 PMC2690996

[43] Dewey CN, Li B. RSEM: accurate transcript quantification from RNA-Seq data with or without a reference genome. Bmc Bioinformatics. 2011;12(1):323. 21816040 10.1186/1471-2105-12-323 PMC3163565

[44] Conesa A, Nueda MJ, Ferrer A, Talón M. MaSigPro: A method to identify significantly differential expression profiles in time-course microarrayexperiments. Bioinformatics. 2006;22(9):1096–102. 10.1093/bioinformatics/btl056 16481333

