# Supplementary material for: Symptoms of systemic lupus erythematosus are diagnosed in leptin transgenic pigs
Source: PLoS Biol. 2018 Aug 31;16(8):e2005354. doi: 10.1371/journal.pbio.2005354 (PMC6147741; doi:10.1371/journal.pbio.2005354)
Supplement: S1 Protocol — (DOCX) [file pbio.2005354.s001.docx]

**Construction of pLeptin-IRES2-AcGFP1**

*Leptin gene harvesting*

Total RNA extracted from the adipose tissue of pigs was used for reverse transcription, and then leptin was amplified using a template and designed primers with the upstream sequence 5’-GGCCCCAGAAGCACATCC-3’ and the downstream sequence 5’-TCAGCAGCCAGGGCTGAG-3’. The annealing temperature was 55°C, and the amplicon length was 533 bp. The product of leptin was detected by agarose gel electrophoresis and then isolated using a DNA Gel Extraction Kit (TransGen Biotech, Beijing, China).

*Generation of pMD18-Leptin construct*

The obtained leptin DNA was cloned into the pMD18-T vector (TaKaRa, Dalian, China) in a 16°C homeothermic metal bath for 2 hours with 10 µl of total reaction solution comprising 3 µl of leptin gene, 1 µl of pMD18-T vector, 1 µl of ddH_2_O and 5 µl of Solution I. After transformation, the recombined plasmid was extracted, identified and named pMD18-Leptin.

*Ligation of pMD18-Leptin and pIRES2-AcGFP1*

pMD18-Leptin and pIRES2-AcGFP1 were digested with SalI and EcoRI at 37°C for 2 hours. Then, the product was electrophoresed in an agarose gel, and the desired fragment was isolated using a Gel Extraction Kit (TransGen Biotech, Beijing, China). Following the digestion, identification and isolation, leptin and the scaffold vector pIRES2-AcGFP1 were ligated at a ratio of 4:1 in a homeothermic metal bath at 16°C for 2 hours in a total reaction volume of 10 µl comprising 4 µl of leptin, 1 µl of pIRES2-AcGFP1 vector and 5 µl of Solution I. After transformation, the recombinant was extracted, identified and named pLeptin-IRES2-AcGFP1. Before transfection into fibroblasts, the vector was digested and linearized with *ApaLI.*

**Preparation of Yorkshire pig fetal fibroblasts**

*Fetal fibroblast culture and passage*

A 30-day Yorkshire pig fetus (♂) was obtained surgically, immediately immersed in DMEM solution with 100 IU/mL penicillin-streptomycin solution (PS) and 10% FBS, and transported to the laboratory. After fetal head, tail, limbs and internal organs were removed under a laminar flow hood, the remaining fetal tissue was washed three times with sterile PBS (including 5% PS) and five times with PBS without PS. The tissue was then cut into pieces with ophthalmic scissors. The fetal tissue was transferred to a T25 flask, 4 mL collagenase IV was added, and the tissue was digested on a horizontal shaker in an incubator at 37°C for 4 hours. After the collagenase was removed by centrifugation, the collected cells were cultured in a 37°C, 5% CO_2_ incubator with DMEM culture medium containing 10% FBS and 1% PS. The culture medium was changed every 3 days. After reaching 80% confluence, the cells were passaged and frozen.

*G418 toxicity susceptibility testing in fibroblasts*

Thawed fibroblasts were seeded into 24-well plates at a density of 5×10^4^ cells/mL and were divided into 10 groups with three repeats. After the cells reached 50-70% confluence, G418 was added to each plate at concentrations of 0 μg/mL, 100 μg/mL, 200 μg/mL, 300 μg/mL, 400 μg/mL, 500 μg/mL, 600 μg/mL, 700 μg/mL, 800 μg/mL and 1000 μg/mL. For two weeks, the culture medium was replaced every two days and supplemented with G418, and the degree of cell death was immediately recorded. The best screening concentration was determined as the one that caused the death of all cells within one week.

**pLeptin-IRES2-AcGFP1-transfected fibroblasts**

The day before transfection with an electroporator (Bio-Rad Gene Pulser Xcell, USA), the fibroblasts were passaged at an appropriate density. After that, adherent cells in the logarithmic growth phase were digested and collected, maintaining a density of 1×10^6^ cells/mL. After the removal of PBS, the cells were suspended with electric shock buffer and 6 µg of pLeptin-IRES2-AcGFP1 plasmid DNA. The cell suspension was placed in a cuvette (0.4 cm) and electroporated with the parameters of square wave, voltage 250 V, 25 ms, and one pulse (Gene Pulser Xcell^TM^, Bio-RAD). Then, the cells were transferred to 6-well plates and non-selective culture medium was added. The culture medium was changed 48 hours later, and the best concentration of G418 for rapid screening was added. Every 2 days, the culture medium was changed. Seven days later, 300 µg/mL G418 was used to maintain screening clones. Positive clones were further identified by PCR and electrophoresis analysis, digested to passage, and frozen for further utilization.

**Nuclear transfer of over-expressing leptin embryos**

*Donor cell preparation*

Frozen transgenic fibroblasts were recovered, and 300 µg/mL G418 was added 24 hours later to maintain screening. One day before the nuclear transfer, the G418 was removed, and the cells were harvested by conventional methods.

*In vitro maturation (IVM) of oocytes*

Porcine ovaries were collected from the Hongteng abattoir (Chenggong Ruide Food Co., Ltd., Kunming, Yunnan Province, China) with permission to use the animal parts for this study. The ovaries were transported to the laboratory in a 0.9% (w/v) NaCl solution at 30-37°C. Oocytes were obtained and cultured using a previously described method[^34^](#_ENREF_34).

*Somatic cell nuclear transfer*

SCNT was performed using IVM oocytes as recipient cytoplasts as previously described[^34^](#_ENREF_34). After being cultured for 38-42 h, oocytes with expanded cumulus cells were briefly treated with 0.1% (w/v) hyaluronidase and denuded of cumulus cells using a finely drawn glass capillary pipette. Oocytes extruding the first polar body with uniform cytoplasm were cultured in NCSU23 medium supplemented with 0.1 μg/mL demecolcine, 0.05 M sucrose and 4 mg/mL BSA for 0.5-1 h. Each reconstructed embryo formed from a single donor cell was inserted into the perivitelline space of an enucleated oocyte using micromanipulation. The reconstructed embryos were fused with a single direct-current pulse of 200 V/mm for 20 μs using an Electro Cell Fusion Generator LF201 (NEPA GENE Co., Ltd., Japan) in fusion medium [0.25 M D-sorbic alcohol, 0.05 mM Mg(C_2_H_3_O_2_)_2_, 20 mg/mL BSA and 0.5 mM HEPES (free acid)]. Then, the reconstructed embryos were cultured for 2 h in PZM-3 and activated with a single pulse of 150 V/mm for 100 ms in an activation medium containing 0.25 M D-sorbic alcohol, 0.01 mM Ca(C_2_H_3_O_2_)_2_, 0.05 mM Mg(C_2_H_3_O_2_)_2_ and 0.1 mg/mL BSA. The reconstructed embryos were equilibrated in PZM-3 supplemented with 5 μg/mL cytochalasin B for 2 h at 38.5°C in a humidified atmosphere with 5% CO_2_, 5% O_2_ and 90% N_2_ (APM-30D, ASTEC, Japan).

*Reconstructed embryo culture*

Reconstructed embryos were cultured in PZM-3 medium and placed in an incubator supplied with 5% CO_2_, 5% O_2_ and 90% N_2_ at 38.5°C in a humidified atmosphere. Cleavage and blastocyst formation were monitored on days 2 and 7, respectively. The cell number of the blastocysts was counted after fixation and Hoechst 33342 staining under a laser scanning confocal microscope (TCS SP5II, LEICA, Germany).

**Embryo transfer**

Crossbred (Large White/Landrace Duroc) gilts were used as surrogate mothers for the cloned embryos as previously described[_ENREF_29](#_ENREF_29)[^34^](#_ENREF_34). Pregnancy was detected approximately 23 days after surgical transfer using an ultrasound scanner (HS-101 V, Honda Electronics Co., Ltd., Yamazuka, Japan).
