# Supplementary figures and images for: Symptoms of systemic lupus erythematosus are diagnosed in leptin transgenic pigs
Source: PLoS Biol. 2018 Aug 31;16(8):e2005354. doi: 10.1371/journal.pbio.2005354 (PMC6147741; doi:10.1371/journal.pbio.2005354)

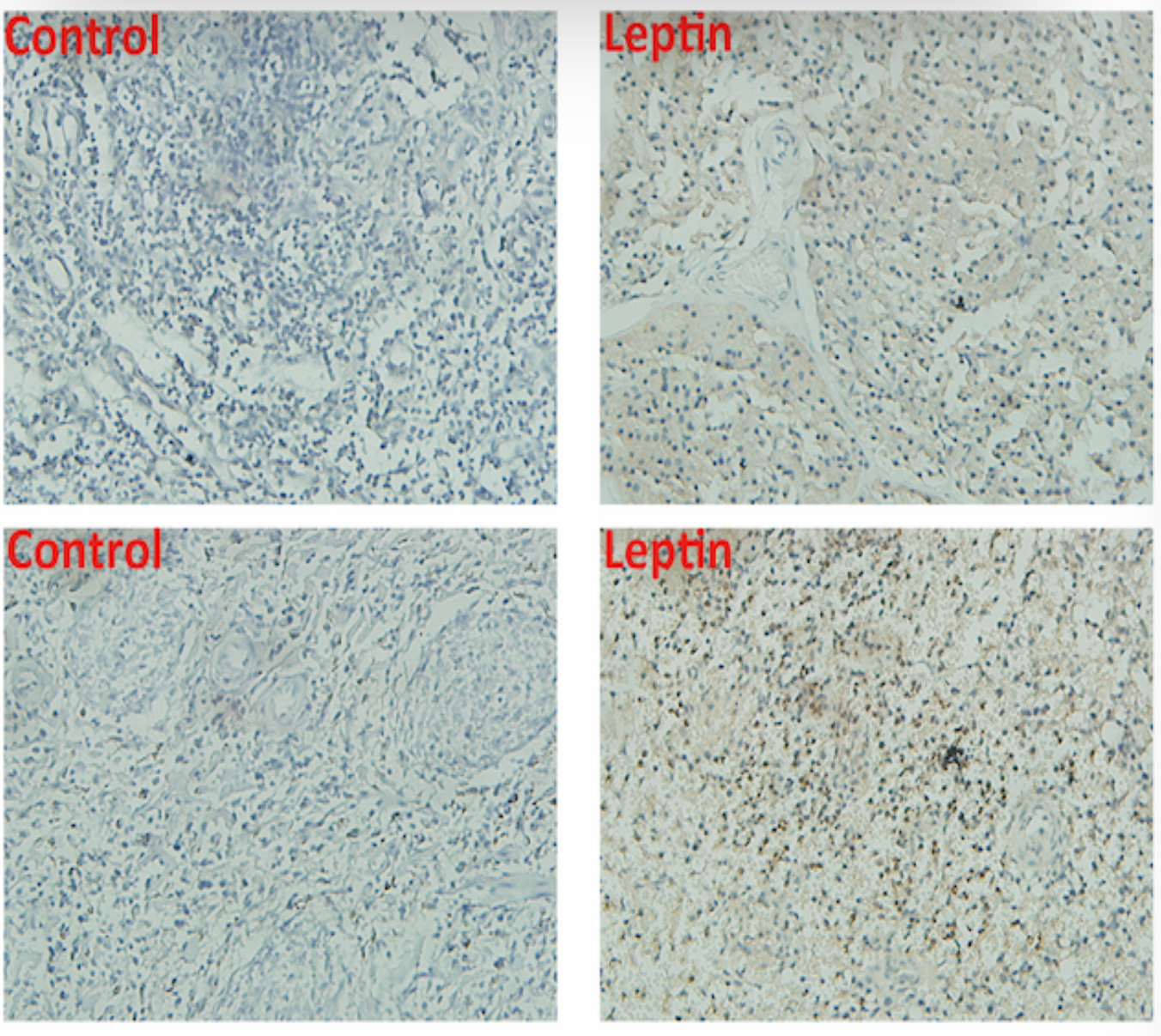

Supplement: S1 Fig — IHC, immunohistochemistry. (TIF) [file pbio.2005354.s002.tif]

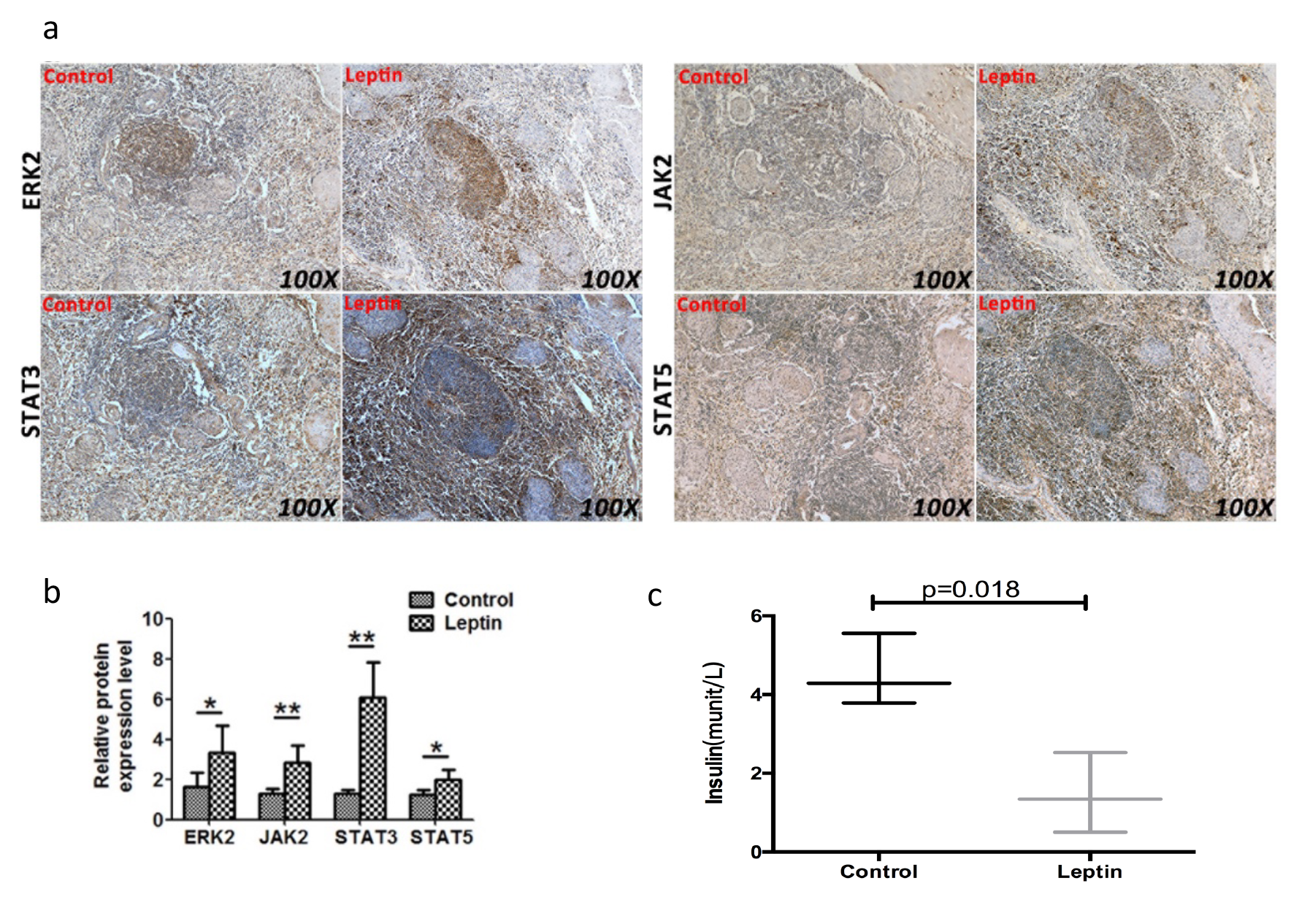

Supplement: S2 Fig — (a) The sections of spleen tissues from both control and transgenic leptin pigs were probed with ERK2, JAK2, STAT3, and STAT5 antibodies. (b) Nine scopes were randomly captured for statistical analysis. (c) Serum insulin levels in pigs. The mean level of signal intensity and standard error bars are presented (*p < 0.05, **p < 0.01). (TIF) [file pbio.2005354.s003.tif]

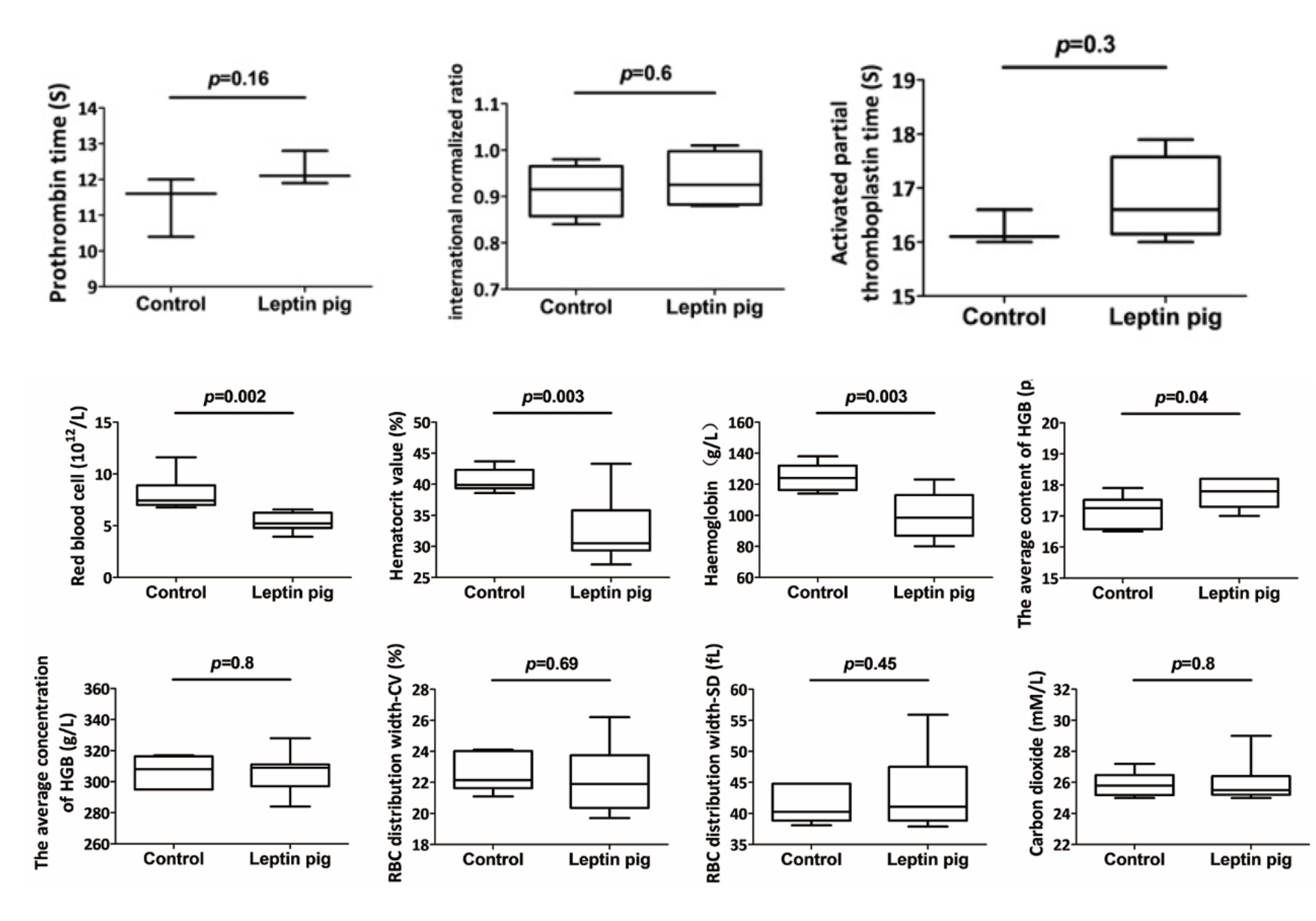

Supplement: S3 Fig — HGB, hemoglobin; RBC, red blood cell. (TIF) [file pbio.2005354.s004.tif]

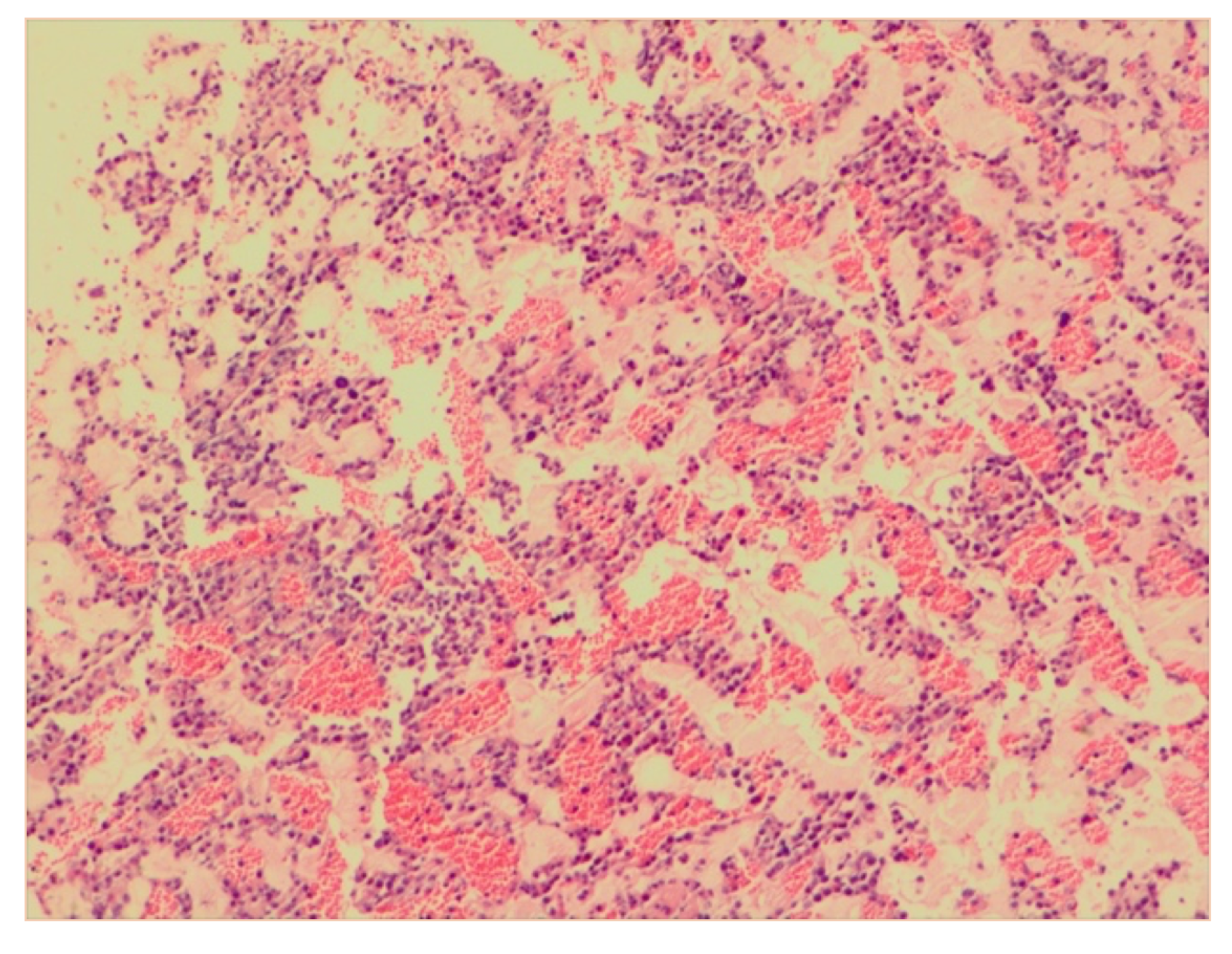

Supplement: S4 Fig — H&E, hematoxylin and eosin. (TIF) [file pbio.2005354.s005.tif]

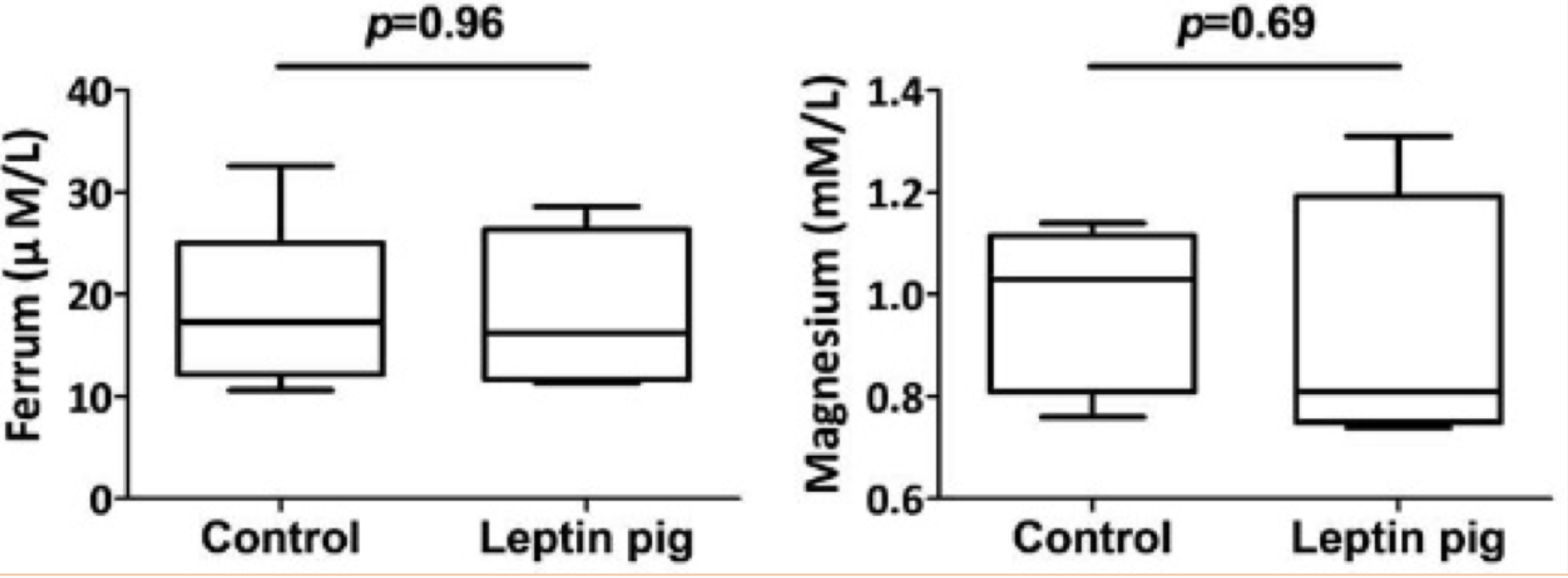

Supplement: S5 Fig — (TIF) [file pbio.2005354.s006.tif]

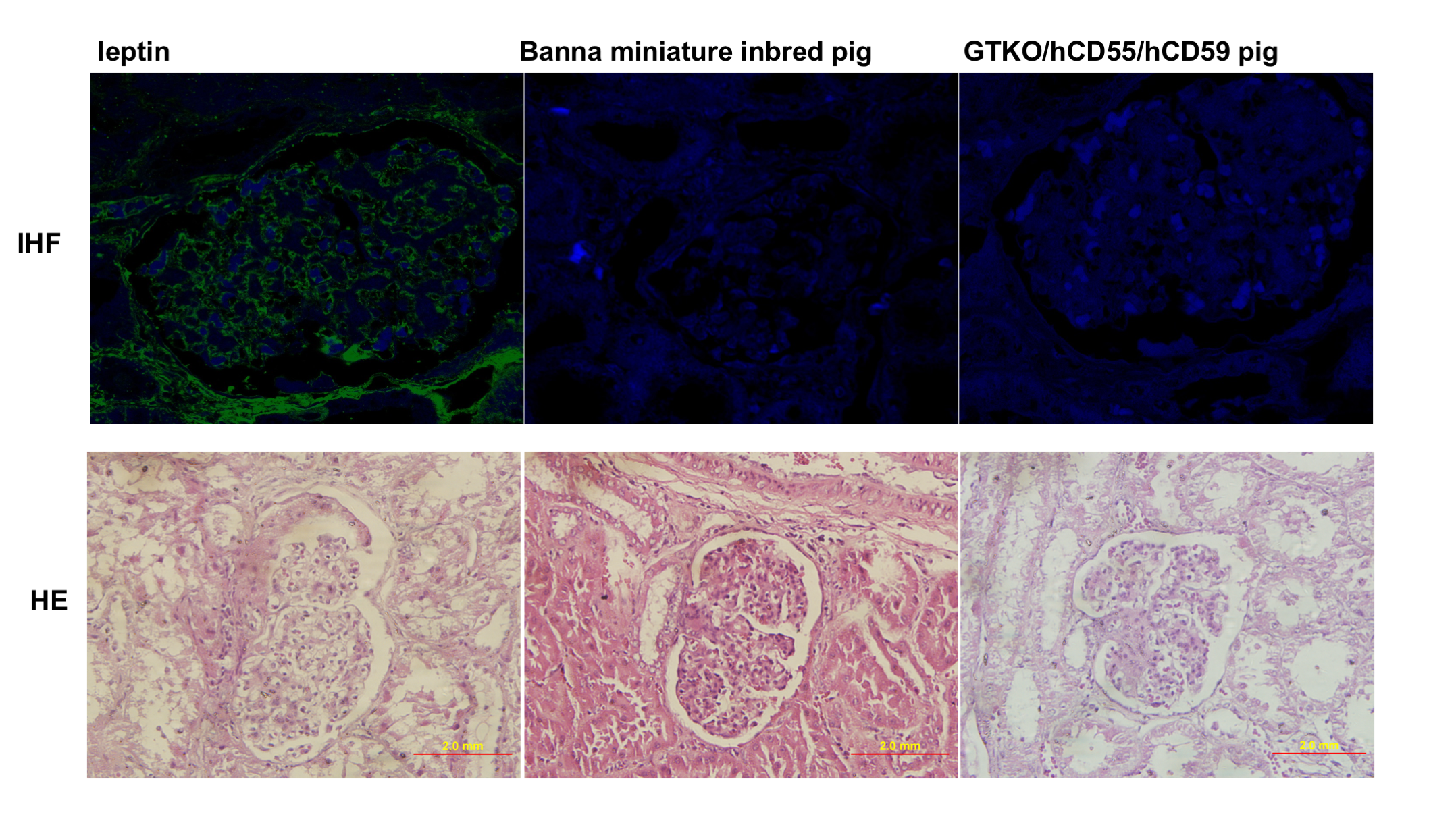

Supplement: S6 Fig — The SCNT technique was used to generate the leptin pigs. H&E, hematoxylin and eosin; Ig, immunoglobulin; IHC, immunohistochemistry; SCNT, somatic cell nuclear transfer. (TIFF) [file pbio.2005354.s007.tiff]

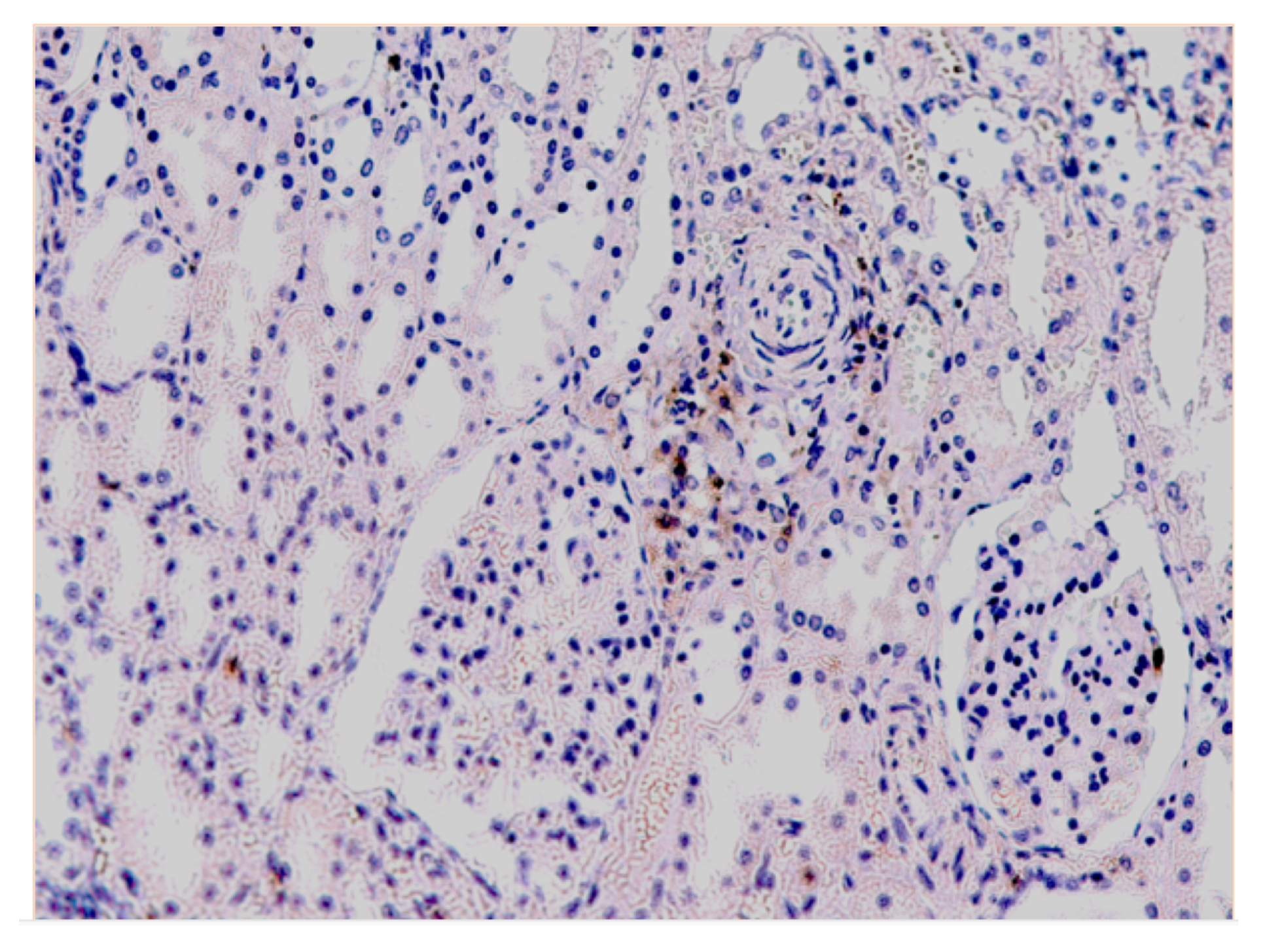

Supplement: S7 Fig — IHC, immunohistochemistry. (TIF) [file pbio.2005354.s008.tif]

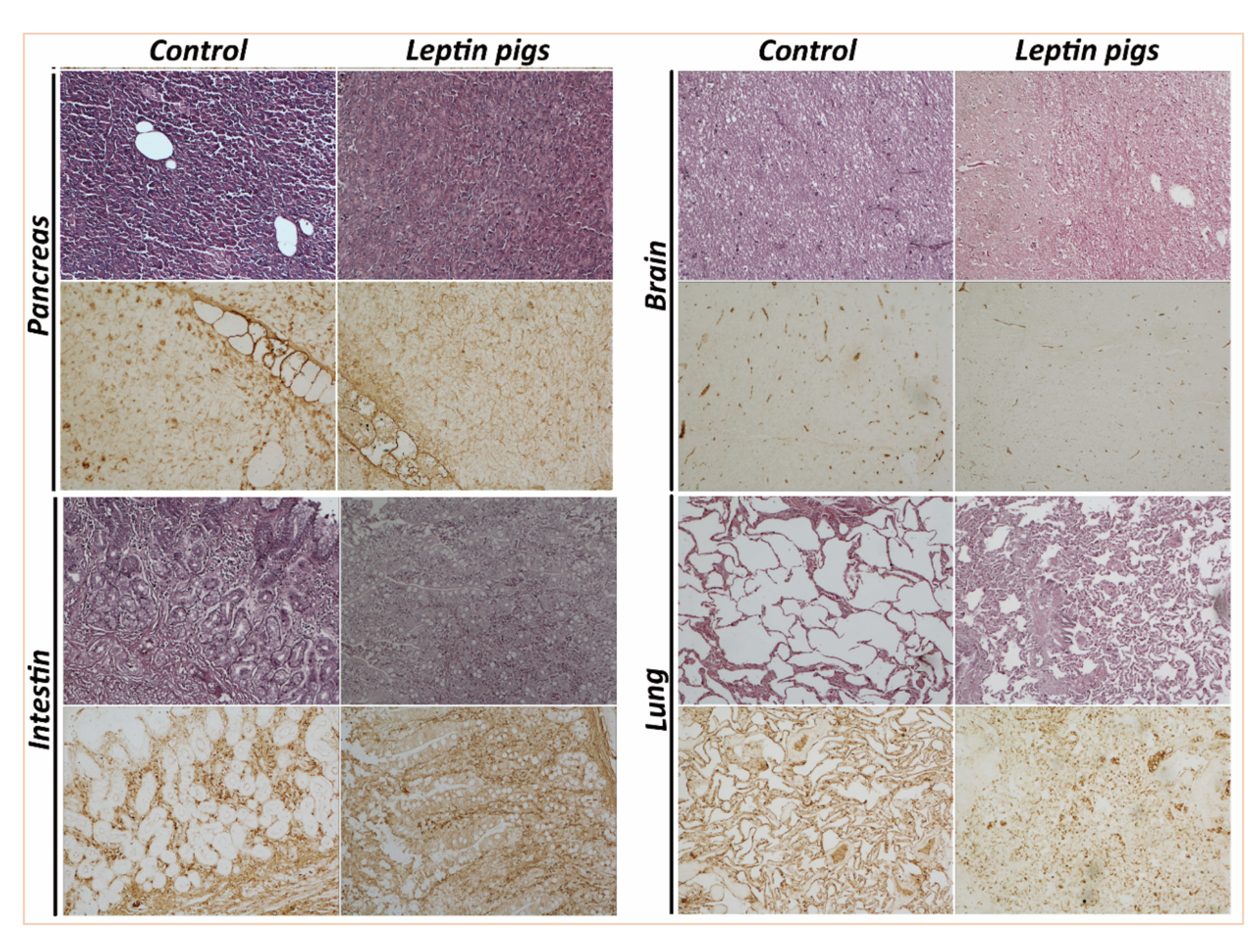

Supplement: S8 Fig — H&E, hematoxylin and eosin; Ig, immunoglobulin; IHC, immunohistochemistry. (TIF) [file pbio.2005354.s009.tif]

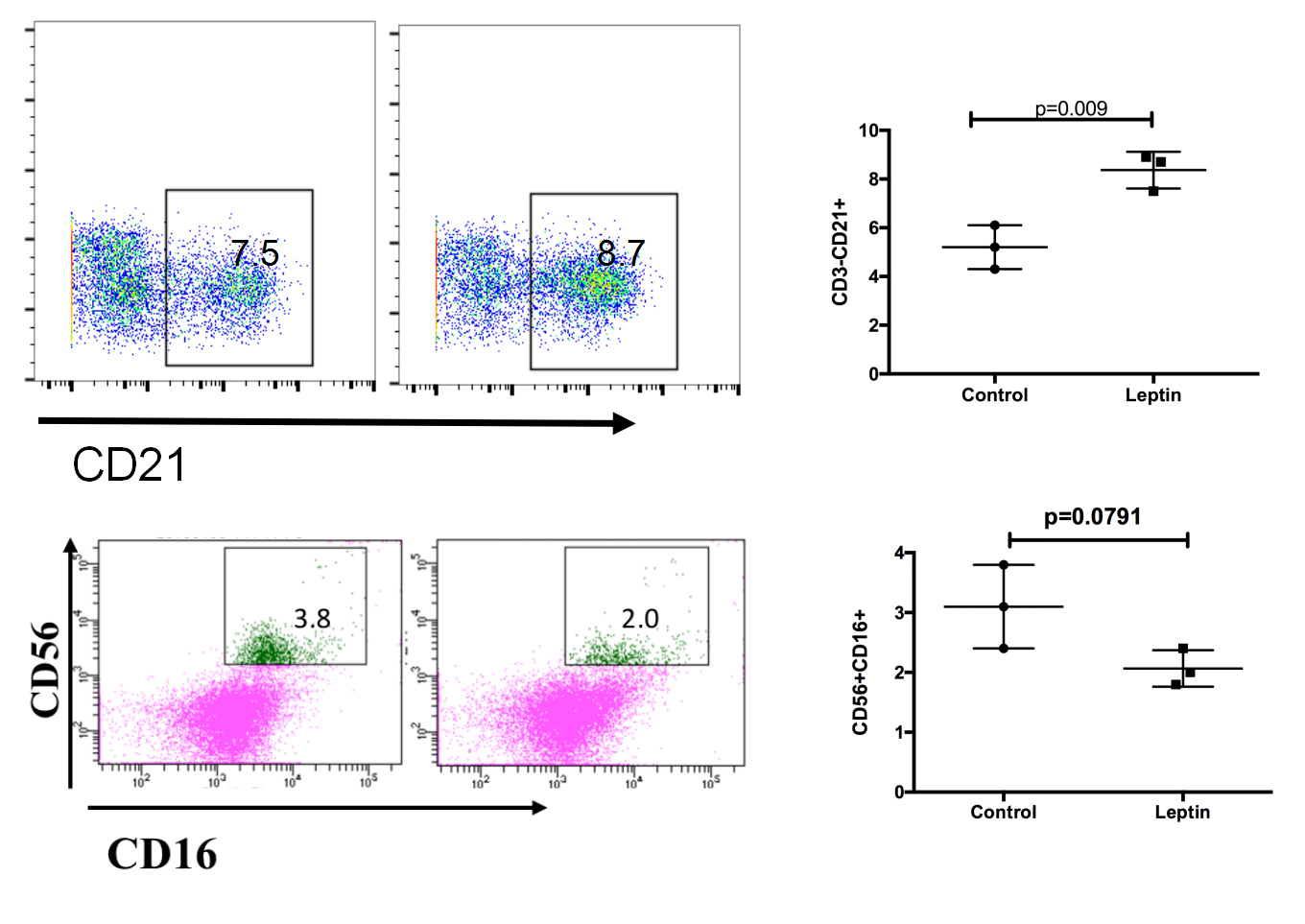

Supplement: S9 Fig — NK, natural killer; PBMC, peripheral blood mononuclear cell. (TIF) [file pbio.2005354.s010.tif]

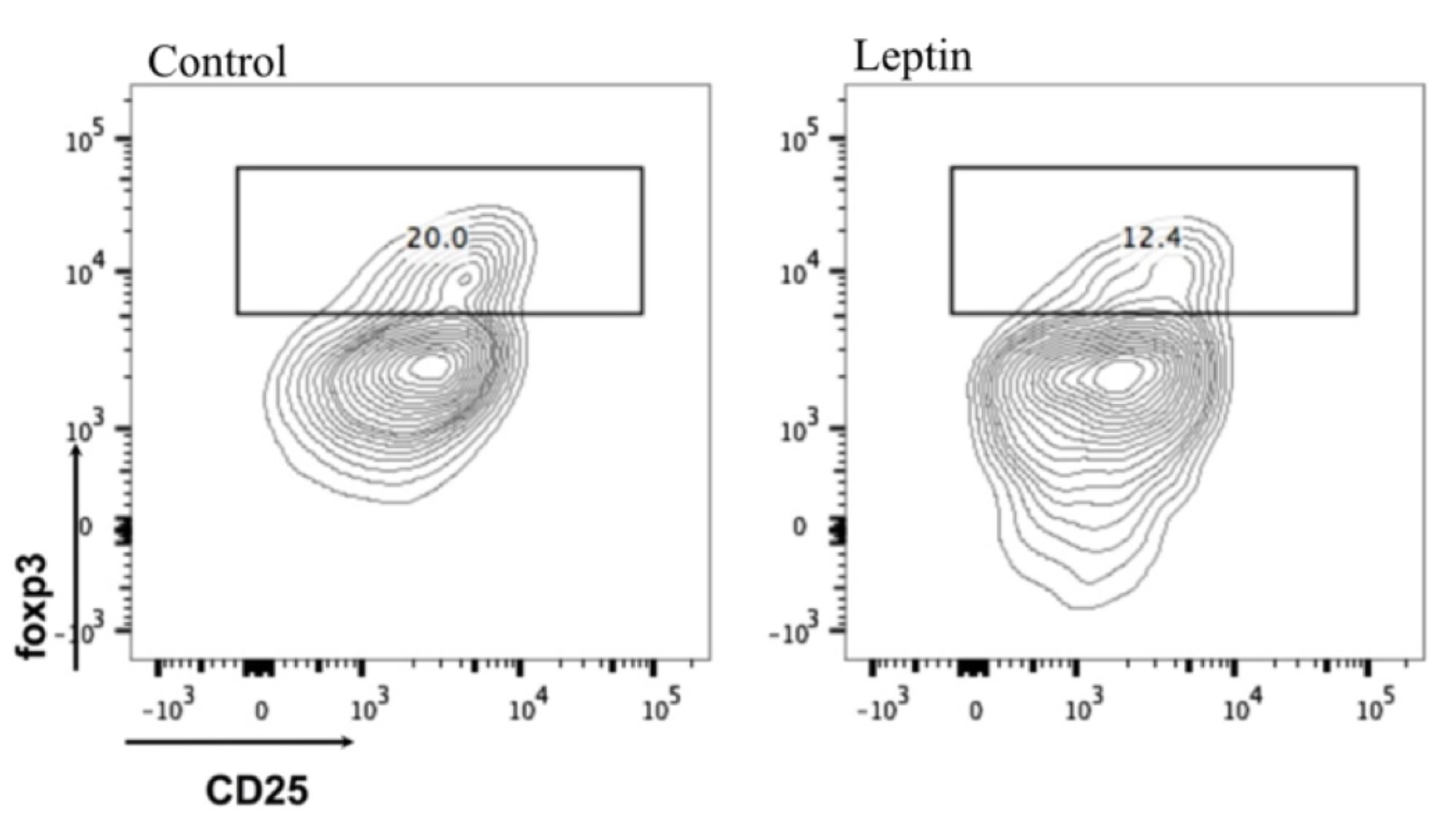

Supplement: S10 Fig — Magnetic-bead–sorted CD4+CD25− T cells were incubated with 2.5 μg/mL anti-CD3/CD28 Ab for 3 d. The culture media for the Tregs contained added TGF-β (0.1 ng/μL), with or without leptin (100 ng/mL). Cells were analyzed by FACS 3 d later. FACS, fluorescence-activated cell sorting; Treg, regulatory T cell. (TIF) [file pbio.2005354.s011.tif]

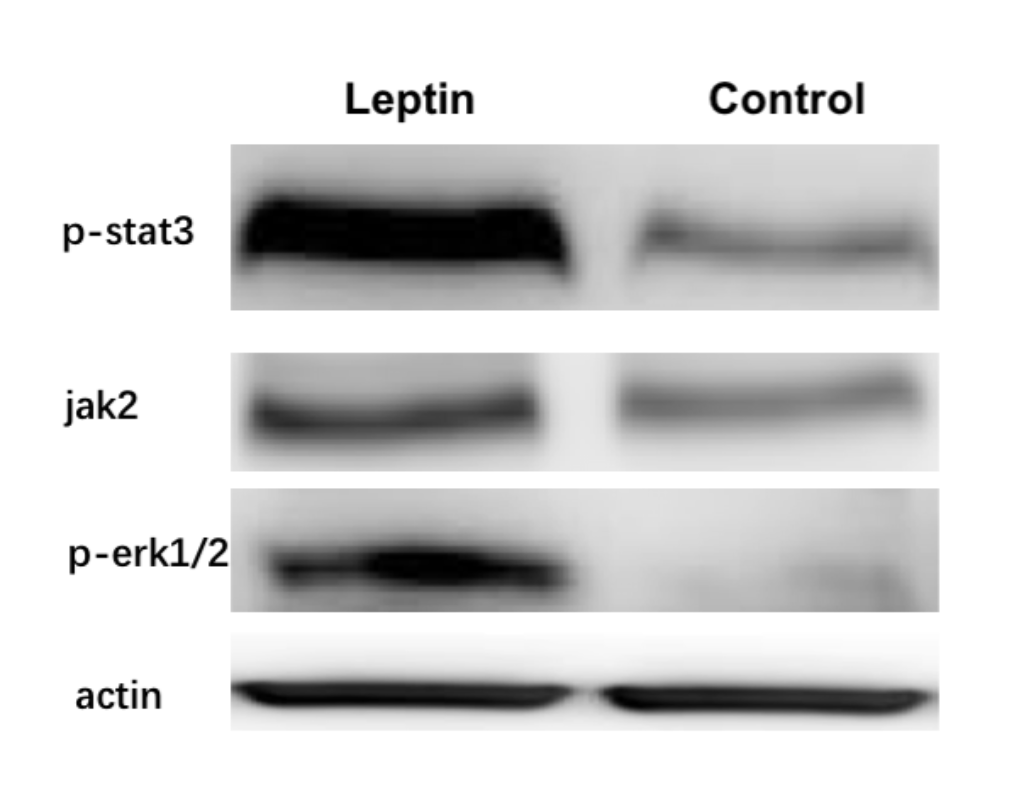

Supplement: S11 Fig — 10% of the lysate was used to detect total levels of the respective proteins. (TIF) [file pbio.2005354.s012.tif]
