# Supplementary material for: Symptoms of systemic lupus erythematosus are diagnosed in leptin transgenic pigs
Source: PLoS Biol. 2018 Aug 31;16(8):e2005354. doi: 10.1371/journal.pbio.2005354 (PMC6147741; doi:10.1371/journal.pbio.2005354)
Supplement: S1 Table — (DOCX) [file pbio.2005354.s013.docx]

S1.Table Oligonucleotides

| Primer name | Primer sequence | Purpose  of the primers | PCR cycles |
| --- | --- | --- | --- |
| pleptin_1F | GGC CCC AGA AGC ACA TCC | pleptin  ORF amplification | 32 |
| pleptin_1R | TCA GCA GCC AGG GCT GAG |  |  |
| pleptin_2F | CTTTGGCCCTATCTGTCCTACGTTG | pleptin  semi-quantitative PCR | 23 |
| pleptin_2R | TCGCCAGGGTCTGGTCCATCT |  |  |
| GFP_F | ACCCTCGTGACCACCCTGACCT | PCR to test  GFP expression | 20 |
| GFP_R | TCTTGTAGTTGCCGTCGTCCTT |  |  |
| pβ-actin_F | ATCAGCAAGCAGGAGTACGACG | PCR to check pig β-actin  as internal reference | 20 |
| pβ-actin_R | GCCATGCCAATCTCATCTCATTTT |  |  |
